# Dual treatment with kynurenine pathway inhibitors and NAD ^+^ precursors synergistically extends life span in *Drosophila*

**DOI:** 10.1111/acel.14102

**Published:** 2024-03-13

**Authors:** Mariann M. Gabrawy, Reyhan Westbrook, Austin King, Nick Khosravian, Neeraj Ochaney, Tagide DeCarvalho, Qinchuan Wang, Yuqiong Yu, Qiao Huang, Adam Said, Michael Abadir, Cissy Zhang, Pratik Khare, Jennifer E. Fairman, Anne Le, Ginger L. Milne, Fernando J. Vonhoff, Jeremy D. Walston, Peter M. Abadir

**Affiliations:** ^1^ School of Medicine, Division of Geriatric Medicine and Gerontology, Biology of Healthy Aging Program Johns Hopkins University Baltimore Maryland USA; ^2^ Department of Biological Sciences University of Maryland, Baltimore County Baltimore Maryland USA; ^3^ Emory University Atlanta Georgia USA; ^4^ University of Maryland, College Park College Park Maryland USA; ^5^ Gigantest Inc. Baltimore Maryland USA; ^6^ Department of Arts as Applied to Medicine Johns Hopkins University Baltimore Maryland USA; ^7^ Vanderbilt University Vanderbilt Brain Institute, Neurochemistry Core Nashville Tennessee USA

**Keywords:** aging, kyneurnine, longevity regulation, NAD, tryptophan

## Abstract

Tryptophan catabolism is highly conserved and generates important bioactive metabolites, including kynurenines, and in some animals, NAD^+^. Aging and inflammation are associated with increased levels of kynurenine pathway (KP) metabolites and depleted NAD^+^, factors which are implicated as contributors to frailty and morbidity. Contrastingly, KP suppression and NAD^+^ supplementation are associated with increased life span in some animals. Here, we used DGRP_229 *Drosophila* to elucidate the effects of KP elevation, KP suppression, and NAD^+^ supplementation on physical performance and survivorship. Flies were chronically fed kynurenines, KP inhibitors, NAD^+^ precursors, or a combination of KP inhibitors with NAD^+^ precursors. Flies with elevated kynurenines had reduced climbing speed, endurance, and life span. Treatment with a combination of KP inhibitors and NAD^+^ precursors preserved physical function and synergistically increased maximum life span. We conclude that KP flux can regulate health span and life span in *Drosophila* and that targeting KP and NAD^+^ metabolism can synergistically increase life span.

## Graphical abstract

Our study explores the impact of kynurenine pathway (KP) modulation and NAD+ supplementation on aging Drosophila. We found that KP inhibition combined with NAD+ precursors significantly enhances physical performance and extends life span. These findings suggest that targeted interventions in KP and NAD+ metabolism can synergistically improve health span and longevity.

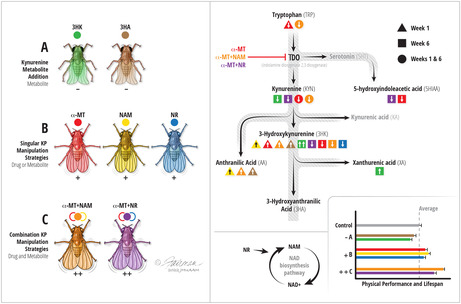

**Abbreviations**
α‐MT: alpha‐methyltryptophan
3‐HAA: 3‐hydroxyanthranilic acid
3‐HK: 3‐hydroxykynurenine
CAFE assay: capillary feeder assay
DGRP: drosophila genetic reference panel
DLM: dorsal longitudinal muscle
IDO: indolamine 2,3‐dioxygenase
IFN‐γ: Interferon gamma
KP: kynurenine pathway
NAD^+^
: nicotinamide adenine dinucleotide
NAM: nicotinamide
NR: nicotinamide riboside
TDO: tryptophan 2,3‐dioxygenase
VNC: ventral nerve cord

## 1 INTRODUCTION

Growing populations of older adults and the increasing prevalence of sarcopenia, physical frailty, and aging‐related diseases highlight the need for novel biological strategies to slow these age‐related declines. Through numerous lines of consistent, consilient evidence, tryptophan metabolism has been linked to life span in metazoans making this pathway an attractive target for interventions which reduce the effects of age. Tryptophan metabolism is highly conserved throughout nature and fluxes of this pathway are linked to longevity in numerous species of both vertebrates and invertebrates (Cervenka et al., 2017).

Tryptophan is an essential amino acid in mammals and insects (Croset et al., 2016; Sang & King, 1961) and is a substrate for synthesizing serotonin, melatonin and several related metabolites collectively known as kynurenines. In mammals, most available tryptophan is degraded through the KP, with the conversion of tryptophan to kynurenine by liver‐specific tryptophan 2,3‐dioxygenase (TDO) being the rate‐limiting step of the pathway (Bender, 1983; Kanai et al., 2009; Liu et al., 2018). One of the major products of the pathway is the energy metabolite and cellular cofactor, nicotinamide adenine dinucleotide (NAD^+^) (Bender, 1983; Canto et al., 2015). In the presence of inflammatory cytokines such as IFN‐γ, indolamine 2,3‐dioxygenase (IDO) is activated and contributes significantly to systemic levels of kynurenines (Alberati‐Giani et al., 1996; Chaves et al., 2001; Fujigaki et al., 2006). Kynurenine 3‐monooxygenase converts kynurenine to 3‐hydroxykynurenine (3‐HK), and inflammatory cytokines also increase its expression and activity (Alberati‐Giani et al., 1996; Chiarugi et al., 2001; Zunszain et al., 2012). In *Drosophila*, major products of the KP include kynurenines and ommochrome pigments.

In humans and flies, elevated levels of kynurenines are noted in a number of aging‐related diseases, including metabolic syndrome (Matsuoka et al., 2016), neurodegenerative disease (Campesan et al., 2011; Schwarcz et al., 2012), and physical frailty (Westbrook et al., 2020). In human serum, metabolites of tryptophan are significantly altered in older subjects compared to young, and the kynurenine/tryptophan ratio is strongly correlated with inflammatory cytokines, frailty status, grip strength, and walking speed (Westbrook et al., 2020). Further, frail older adults with increased vulnerability have elevated serum levels of 3‐HK compared to both non‐frail and young subjects. In combination with studies that identified toxic effects of downstream KP metabolites on neurons and other tissues (Parrott et al., n.d.; Pearson & Reynolds, 1991; Westbrook et al., 2020; Wolfensberger et al., 1983), these findings suggest kynurenines are a causal link between inflammation and the development of aging phenotypes.

Contrastingly, reduced dietary tryptophan and the suppression of KP metabolism are linked to increased longevity. Liver kynurenine/tryptophan ratio correlates negatively with maximum life span in 26 mammalian species (Ma et al., 2015). Limiting dietary tryptophan to 30%–40% of controls extends life span in rats (Ooka et al., 1988; Segall & Timiras, 1976) and mice (De Marte & Enesco, 1986); however, kynurenines were not measured in these early studies. RNAi depletion of TDO inhibits the KP and extends life span in *C. elegans* (van der Goot et al., 2012). Both genetically and pharmacologically suppressing the KP extends life span in *Drosophila* (Oxenkrug, 2010; Oxenkrug et al., 2011).

Manipulating NAD^+^ levels can also influence life span in metazoans. In *Drosophila*, whole‐body overexpression of nicotinamidase (Balan et al., 2008) and nicotinamide mononucleotide supplementation (Fang, Hou, Lautrup, et al., 2019) increased NAD^+^ levels and extended life span. Dietary supplementation of the NAD^+^ precursors nicotinamide (NAM) and nicotinamide riboside (NR) can extend life span in *C. elegans* (Mouchiroud et al., 2013). Chronic NAM supplementation improves health span measures in mice without extending life span (Mitchell et al., 2018). However, studies examining the effects of NAD^+^ precursors in *Drosophila* are lacking.

**FIGURE 1:**
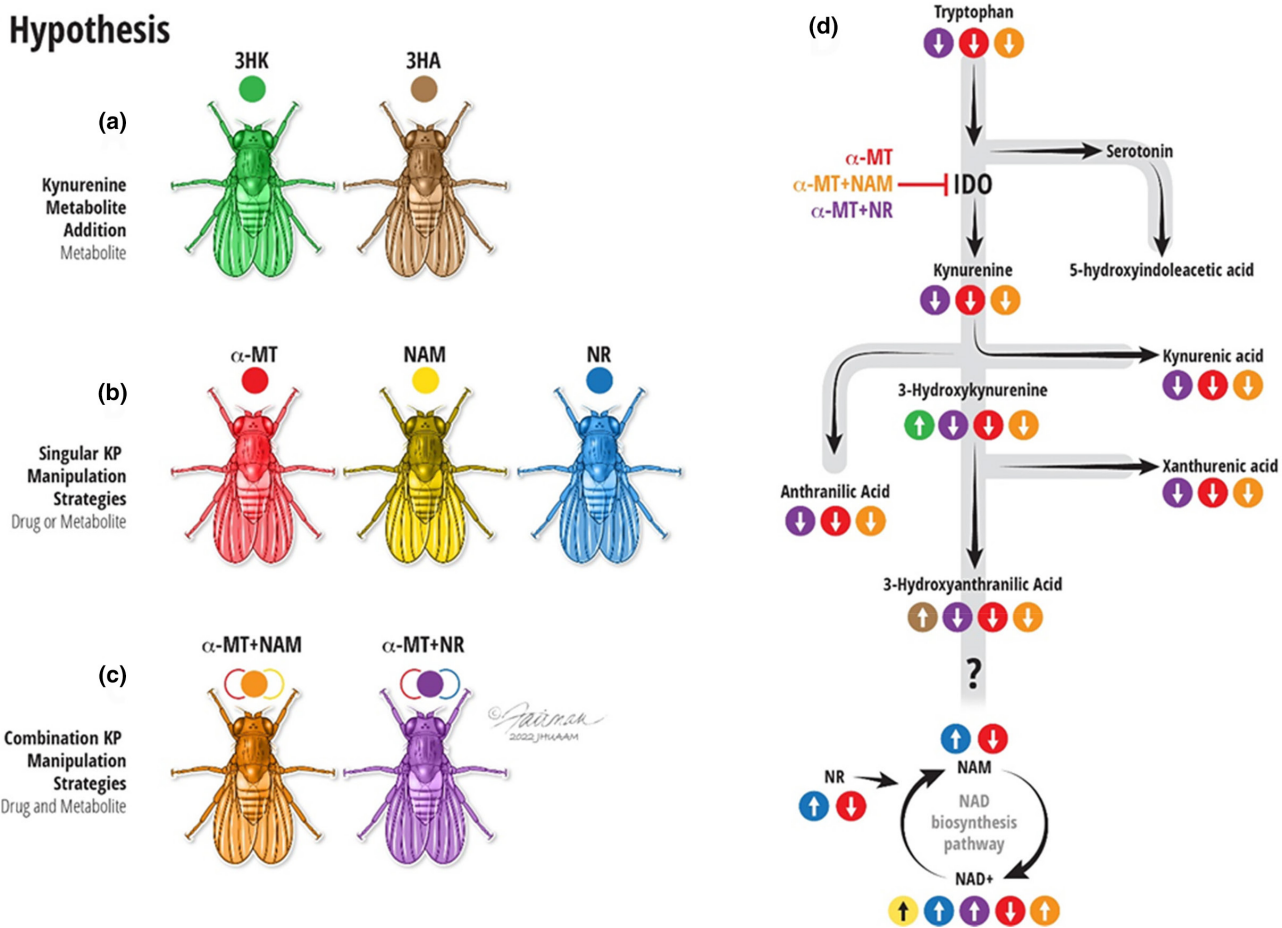
A conceptual framework for kynurenine pathway manipulation strategies. We used DGRP_229 flies to determine the effects of manipulating KP metabolites on physical performance, life span, and dendritic anatomy. To model elevations in potentially deleterious kynurenines, we fed flies 3‐HK and 3‐HAA (a). To examine the effects of KP blockade or NAD^+^ precursor supplementation, we fed flies either alpha‐methyl tryptophan (α‐MT), nicotinamide (NAM), or nicotinamide riboside (NR) (b). To test whether administering KP blockade in tandem with NAD^+^ precursor supplementation elicited any additional benefit, we fed flies combinations of α‐MT + NAM or α‐MT + NR (c). The anticipated effects of treatments on KP metabolites are depicted by arrows on the pathway (d).

Here, we set out to determine the effects of chronically (1) elevating levels of downstream KP metabolites, (2) suppressing KP metabolism, (3) supplementing with NAD^+^ precursor, or (4) simultaneously suppressing KP metabolism and supplementing with NAD^+^ precursors (see schematic in Figure 1), on age‐related changes in physical performance and life span. Based on many independent consilient observations, we hypothesized that elevating levels of downstream kynurenines will decrease physical performance and life span and that suppressing the production of kynurenines while supplementing with NAD^+^ precursors will reduce the effects of age via KP suppression or NAD^+^ precursor supplementation, individually.

## 2 RESULTS

### 2.1 Combination treatment improves physical performance in old age

We first examined the effects of aging on physical performance (Figure S1) as described in Gabrawy et al. (2019). Aging caused a significant decrease in climbing speed (Figure S1A) and endurance (Figure S1B) at every time point. Failure rates of climbing speed (Figure S1C) and endurance (Figure S1D) tests also increased with age.

**FIGURE 2:**
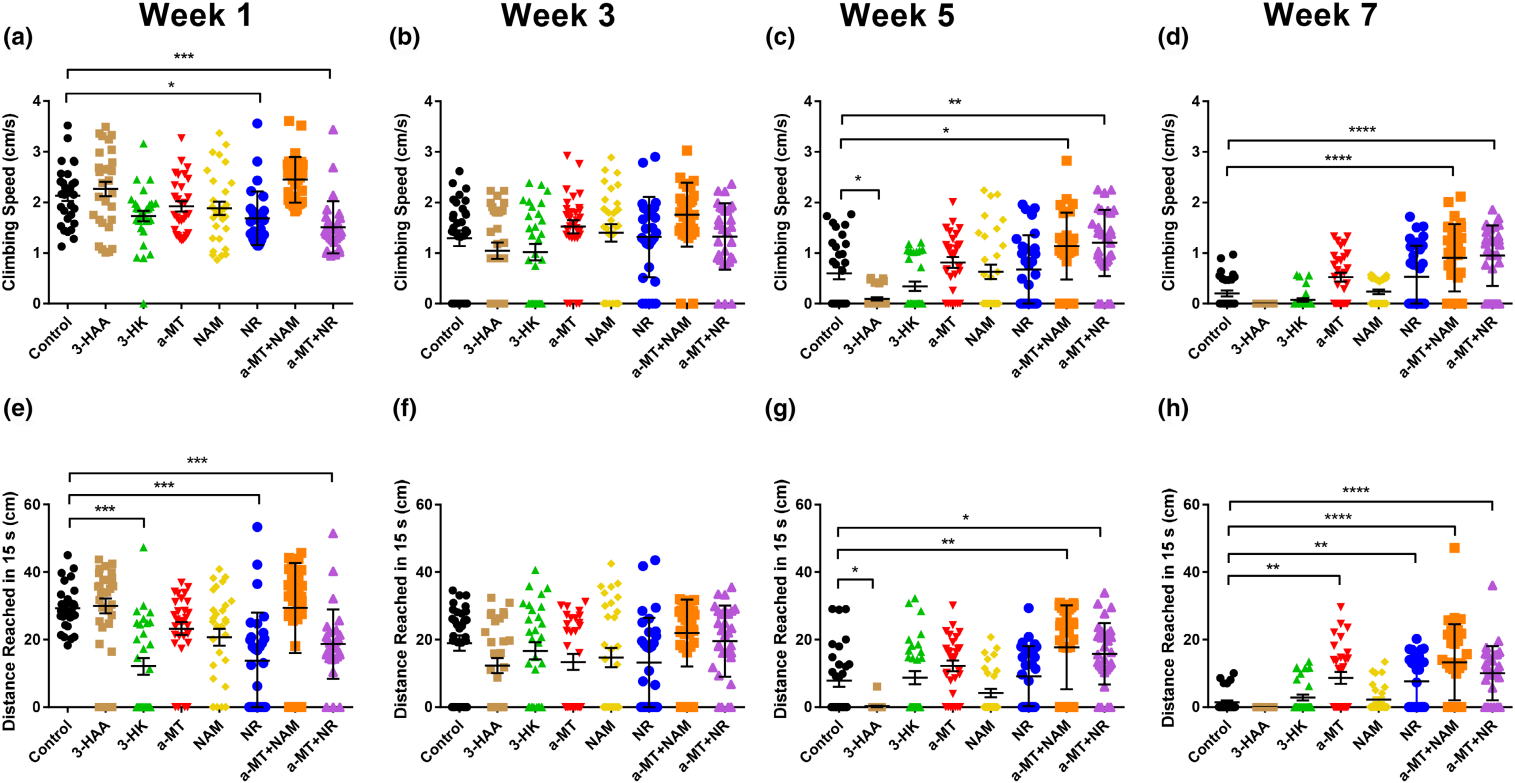
The effects of KP and NAD^+^ salvage pathway manipulation on physical performance of DGRP_229 flies. Climbing speed (a–d) and Endurance (e–h) were measured at 1, 3, 5, and 7 weeks of age (*n* = 30 per treatment group). Significance was determined using the Kruskal–Wallis one‐way ANOVA with Dunn's multiple comparisons test. *p < 0.05, **p < 0.01, ***p < 0.001, ****p < 0.0001.

We then examined the effects of manipulating components of the KP and NAD^+^ salvage pathways (as described in Figure 1) on physical performance. At 1 week (young age), only flies treated with NR and α‐MT + NR had significantly different climbing speeds compared to the control group. In both cases, the climbing speed decreased with treatment (Figure 2a). At Week 3 (middle age), no treatments were significantly different from the control group (Figure 2b). At Week 5 (mid‐old age), flies treated with 3‐HAA were significantly slower than controls, while α‐MT + NAM and α‐MT + NR treated flies had significantly faster climbing speeds (Figure 2c). At Week 7 (old age), the combination‐treated flies had significantly faster‐climbing speed than the control flies (Figure 2d).

Endurance, defined as the distance (cm) climbed in 15 s at young age, was significantly decreased when flies were treated with 3‐HK, NR, and α‐MT + NR (Figure 2e). However, in middle age, there were no significant differences between any of the treatment groups (Figure 2f). At middle‐old age, flies treated with 3‐HAA had significantly decreased endurance while there was a significant increase in endurance for the α‐MT + NAM and α‐MT + NR treatment groups relative to the control group (Figure 2g). At old age, flies treated with α‐MT + NAM, α‐MT + NR, α‐MT, and NR had increased endurance compared to the control group (Figure 2h).

### 2.2 Combination treatments reduce failure rate in old age

**FIGURE 3:**
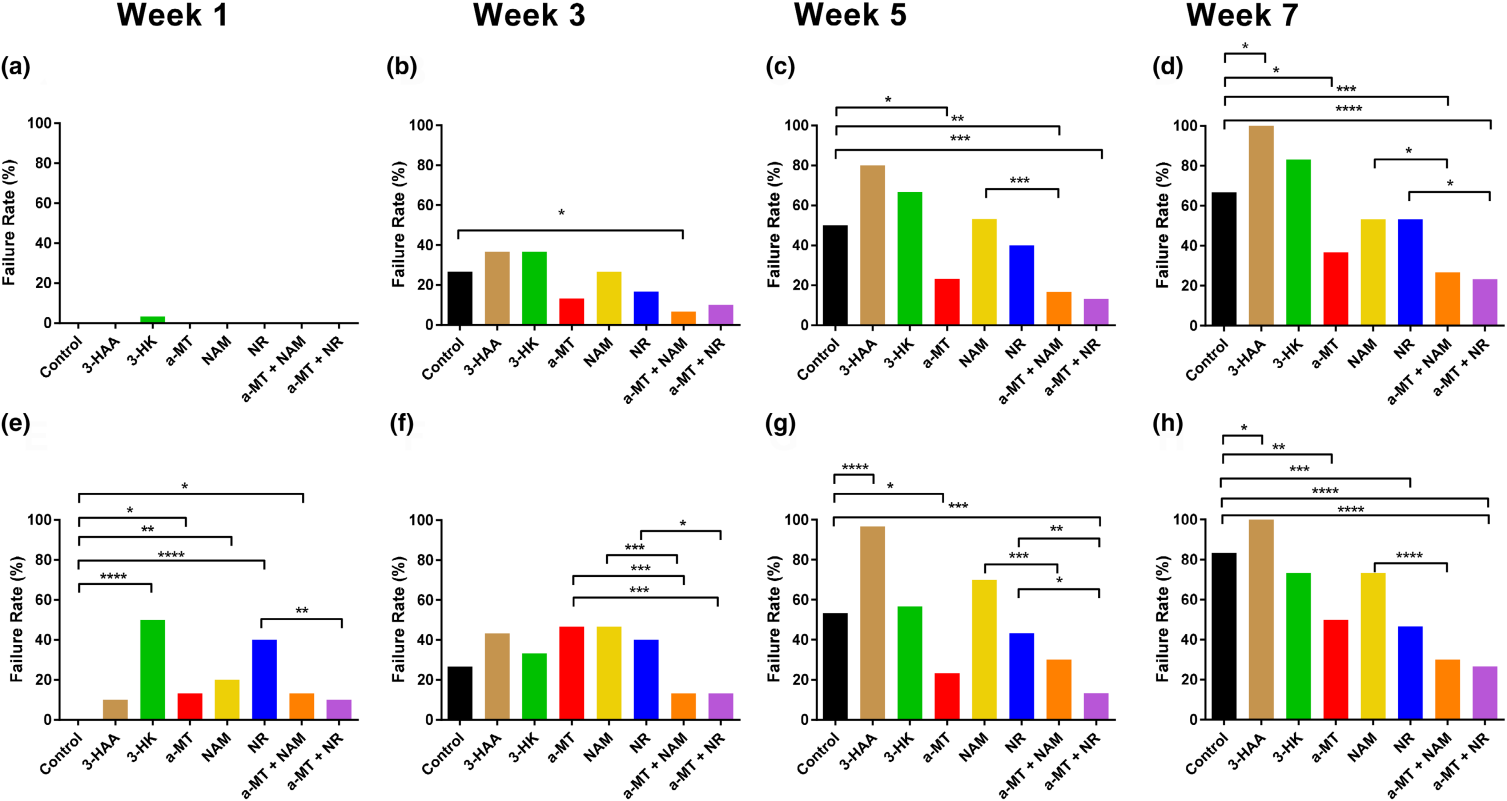
The effects of KP and NAD^+^ salvage pathway manipulation on failure rates of DGRP_229 flies. During the climbing speed test (a–d) and the endurance test (e–h) measured at 1, 3, 5, and 7 weeks of age. Climbing speed failure rate data were measured as the number of flies unable to complete the 9 cm distance due to stopping or falling. The endurance failure rate was calculated as the number of flies unable to complete the 27 cm distance due to stopping or falling (*n* = 30 per treatment group). Significance was determined with Fisher's exact test. *p < 0.05, **p < 0.01, ***p < 0.001, ****p < 0.0001.

Climbing speed failure rate data were measured as the number of flies unable to complete the 9 cm distance due to stopping or falling. This categorical data represents the percentage of flies that either pass or fail each test and is represented as a percentage in each figure. At young age, none of the treatments significantly affected the failure rate, with only 3‐HK having any failures (Figure 3a). Overall, the failure rates of all groups increased in middle age; however, only the α‐MT + NAM treatment significantly reduced the failure rate compared to controls (Figure 3b). At middle‐old age, there was an overall increase in failure rate. We observed a significant decrease in failure rate between the control group and α‐MT, α‐MT + NAM, and α‐MT + NR treated files. Flies treated with 3‐HK, NAM, and NR were not significantly different from the controls and flies fed 3HAA had significantly more fail than the control (Figure 3c). Notably, the flies treated with a combination of α‐MT + NAM or α‐MT + NR had significantly fewer failures than flies treated with NAM or NR alone and numerically fewer failures than α‐MT alone. This pattern of significant differences was also observed at old age, but the differences between the control group and the combination treatments became more pronounced (Figure 3d).

The endurance failure rate was calculated as the number of flies unable to complete the 27 cm distance due to stopping or falling. The failure rate in young flies was significantly elevated in most treatments compared to controls except for 3‐HAA and α‐MT + NR (Figure 3e). While flies in middle age had an overall increase in failure rates for all treatments, there was no significant difference between the control group and any of the treatments. There were notable significant differences between treatments; the combination treatments had reduced failure rates compared to each treatment alone (Figure 3f). Middle‐old age flies treated with 3‐HAA had a significantly increased failure rate compared to controls, while flies treated with α‐MT + NR had a significantly reduced failure rate compared to controls (Figure 3g). Flies at old age showed a significant reduction in failure rates compared to controls when treated with α‐MT, NR, and α‐MT + NR (Figure 3h).

### 2.3 Combination treatments synergistically increase survivorship and life span

**FIGURE 4:**
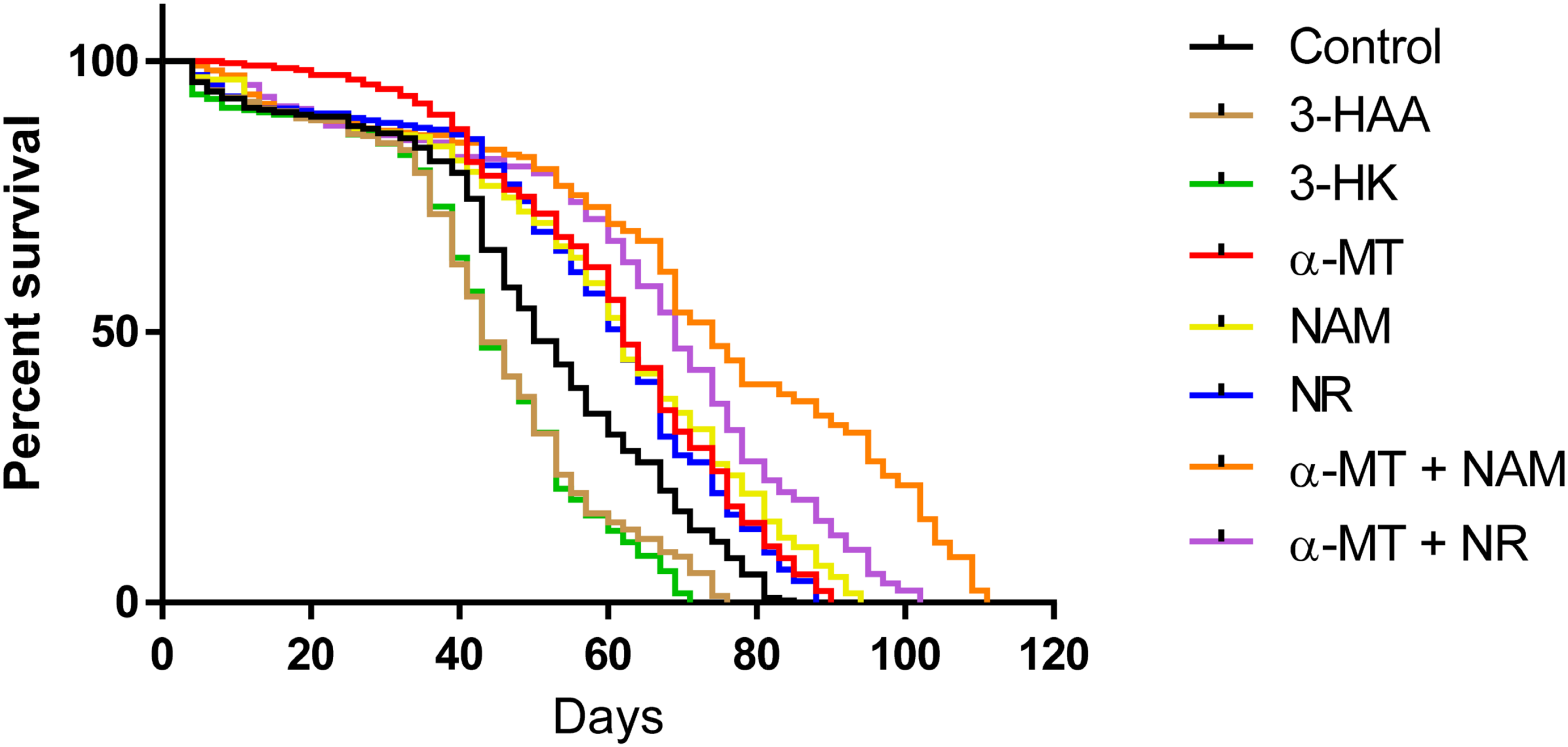
The effects of treatment on survivorship of DGRP_229 (*n* = 250 per treatment group). Significance was determined using Cox regression proportional hazards models.

We measured the life span of virgin males from DGRP_229 reared under control conditions (untreated) or treated with 3‐HK, 3‐HAA, α‐MT, NAM, NR, and combination treatments of α‐MT + NAM and α‐MT + NR (Figure 4, Table S1). Treatment with 3‐HAA and 3‐HK both resulted in significant decreases in average life span duration in comparison with controls with mean durations of 15% and 17% lower, respectively (Table S1A,B).

In contrast, treatment with α‐MT, NAM, NR, and combination treatments of α‐MT + NAM and α‐MT + NR all showed significant increases in average life span compared to controls (Figure 4, Table S1A). The greatest increase in average life span was observed in the combination treatments, with an average life span 30% higher in α‐MT + NAM when compared to the control and 22% higher in α‐MT + NR (Table S1B).

The combination treatment α‐MT + NAM resulted in a significant increase in average life span compared to the individual metabolite treatments α‐MT and NAM (Figure 4, Table S1A), with an average life span 15% longer than that of flies treated with α‐MT alone and 18% longer than that of flies treated with NAM alone (Table S1B). α‐MT + NR also displayed a similar increase in average life span in comparison with treatment with α‐MT and NR individually (Figure 4, Table S1A), with the average life span being 6% longer in comparison with flies treated with α‐MT and 11% longer than flies treated with NAM (Table S1B). This indicates that blockade of the KP via the chronic treatment of exogenous α‐MT paired with supplementation of NAM or NR leads to an optimum increase in life span, whereas chronic treatment with exogenous 3‐HK and 3‐HAA causes life span to decrease. To eliminate food consumption as a confounding factor in these treatments, we tested whether treatments have an effect on feeding rate using the CAFE assay (Ja et al., 2007) and found that treatment has no effect on the feeding rate of male DGRP_229 flies (Figure S2).

We also calculated the effects of treatment on the maximum life span of virgin males from DGRP_229 reared under control conditions (untreated) or treated with 3‐HK, 3‐HAA, α‐MT, NAM, NR, and combination treatments of α‐MT + NAM and α‐MT + NR (Figure S3). While no treatment significantly decreased maximum life span, treatments with NAM, α‐MT + NAM, and α‐MT + NR all significantly increased maximum life span compared to controls. Notably, combination treatments of α‐MT + NAM and α‐MT + NR synergistically extended the maximum life span beyond that of the flies treated with the singular components α‐MT, NAM, or NR (Figure S3).

### 2.4 Combination treatment increases dendrite density

**FIGURE 5:**
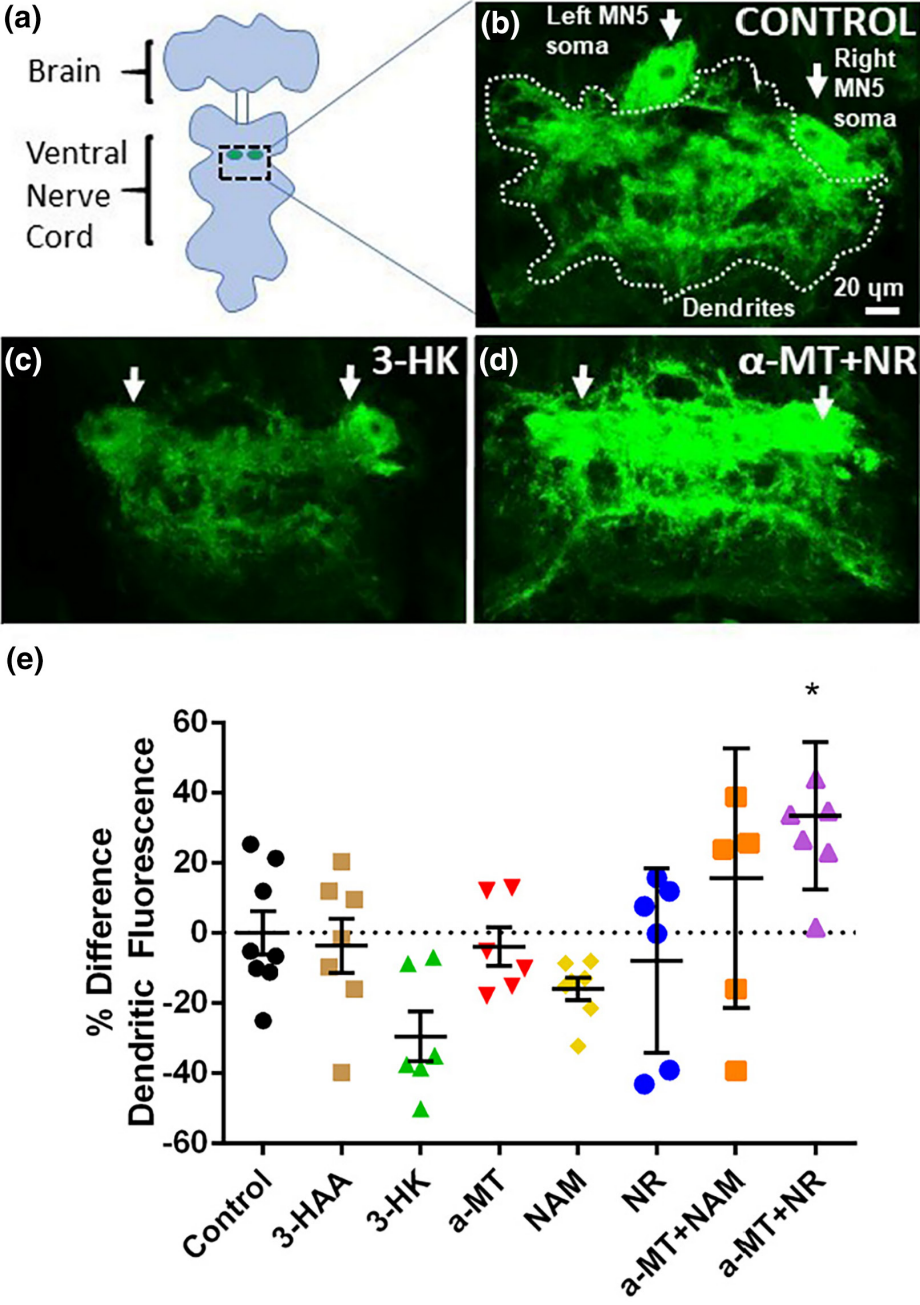
Treatment with kynurenines affects changes in dendritic anatomy. (a) Schematic showing the brain and ventral nerve cord of an adult fly as well as the location of motoneurons that innervate the DLM muscle. Green circles represent cell bodies of the identified motoneuron MN5. Dotted square indicates area of pictures shown in b–d and h–k. (b–d) Projection view images from confocal stacks showing the left and right cell bodies of motoneuron MN5 and the area comprising dendrites from all 10 DLM motoneurons (dotted line) at baseline in flies treated with (b) control food or (c) food supplemented with 3‐HK or (d) α‐MT + NR. (e) Quantification of dendritic fluorescence as calculated by the percentage change of baseline fluorescent value in flies after treatment (*n* = 6–8 per treatment group). Error bars represent SEM.

We used the well‐characterized dendritic arbors of the DLM motoneurons located on the adult ventral nerve cord (VNC) (Figure 5) to assess the effects of kynurenines on dendritic anatomy. As previously described (Mishra‐Gorur et al., 2014), we quantified the relative fluorescent intensity of the dendritic field in controls and in treated flies to test for changes in dendritic anatomy and gain insight into the metabolic health of these dendrites. Flies fed with the combination of α‐MT + NR show a significant increase in dendritic fluorescence as compared to control flies, suggesting that α‐MT + NR treatment may reduce aging‐dependent dendritic degeneration and stabilize dendritic structures. Treatments with 3‐HK and other metabolites lead to numeric but not significant decreases in dendritic fluorescence.

### 2.5 Aging alters kynurenine pathway metabolites

**FIGURE 6:**
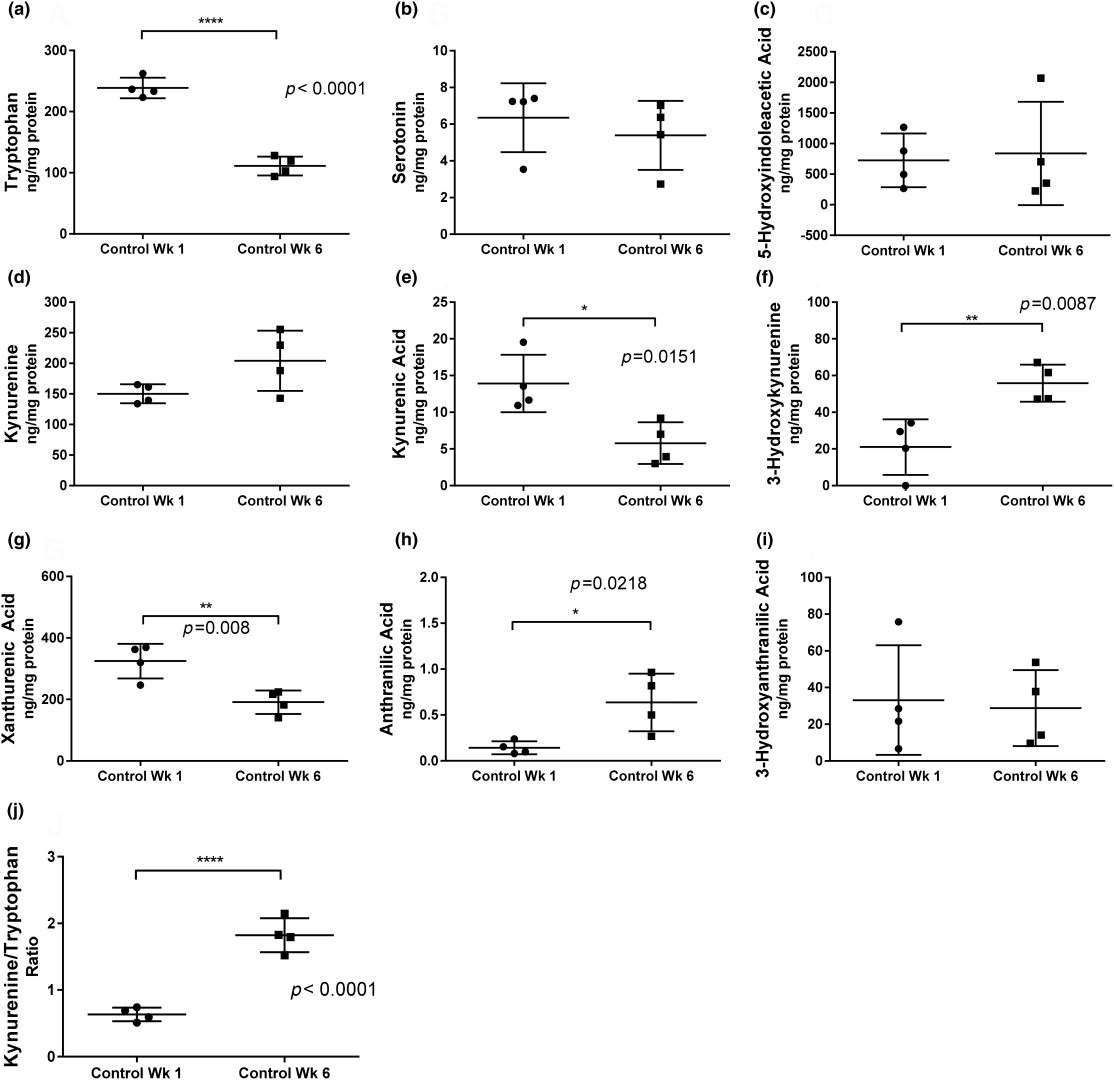
The effects of aging on systemic levels of kynurenines and other tryptophan‐related metabolites in DGRP_229 *Drosophila*. Systemic levels of tryptophan, serotonin pathway, and kynurenine pathway metabolites were measured in flies after 1 week and 6 weeks of age (*n* = 4 samples per treatment group with each sample representing seven to eight flies). We used a targeted metabolomic approach to measure tryptophan (a), serotonin (b), 5‐hydroxyindoleacetic acid (c), kynurenine (d), kynurenic acid (e), 3‐hydroxykynurenine (f), xanthurenic acid (g), anthranilic acid (h), 3‐hydroxyanthranilic acid, (i) and the kynurenine to tryptophan ratio (j). Data were analyzed using the *t*‐test. *p < 0.05, **p < 0.01, ***p < 0.001, ****p < 0.0001.

We measured the effect of age on systemic levels of tryptophan, serotonin pathway, and kynurenine pathway metabolites in DGRP_229 flies (Figure 6a–i). Tryptophan levels were significantly decreased with age (Figure 6a), while serotonin and 5‐hydroxyindoleacetic acid were not significantly altered (Figure 6b,c). Kynurenine was numerically elevated, but the difference was not significant (*p* = 0.055) (Figure 6d). Kynurenic acid levels were significantly decreased in older flies (Figure 6e). 3‐hydroxykynurenine was significantly elevated with age (Figure 6f). Xanthurenic acid was decreased with age (Figure 6g) while anthranilic acid was increased with age (Figure 6h), and 3‐hydroxyanthranilic acid was not significantly changed with age (Figure 6i). The kynurenine to tryptophan ratio was also significantly elevated in older flies (Figure 6j).

### 2.6 Kynurenine pathway manipulation alters levels of key metabolites

**FIGURE 7:**
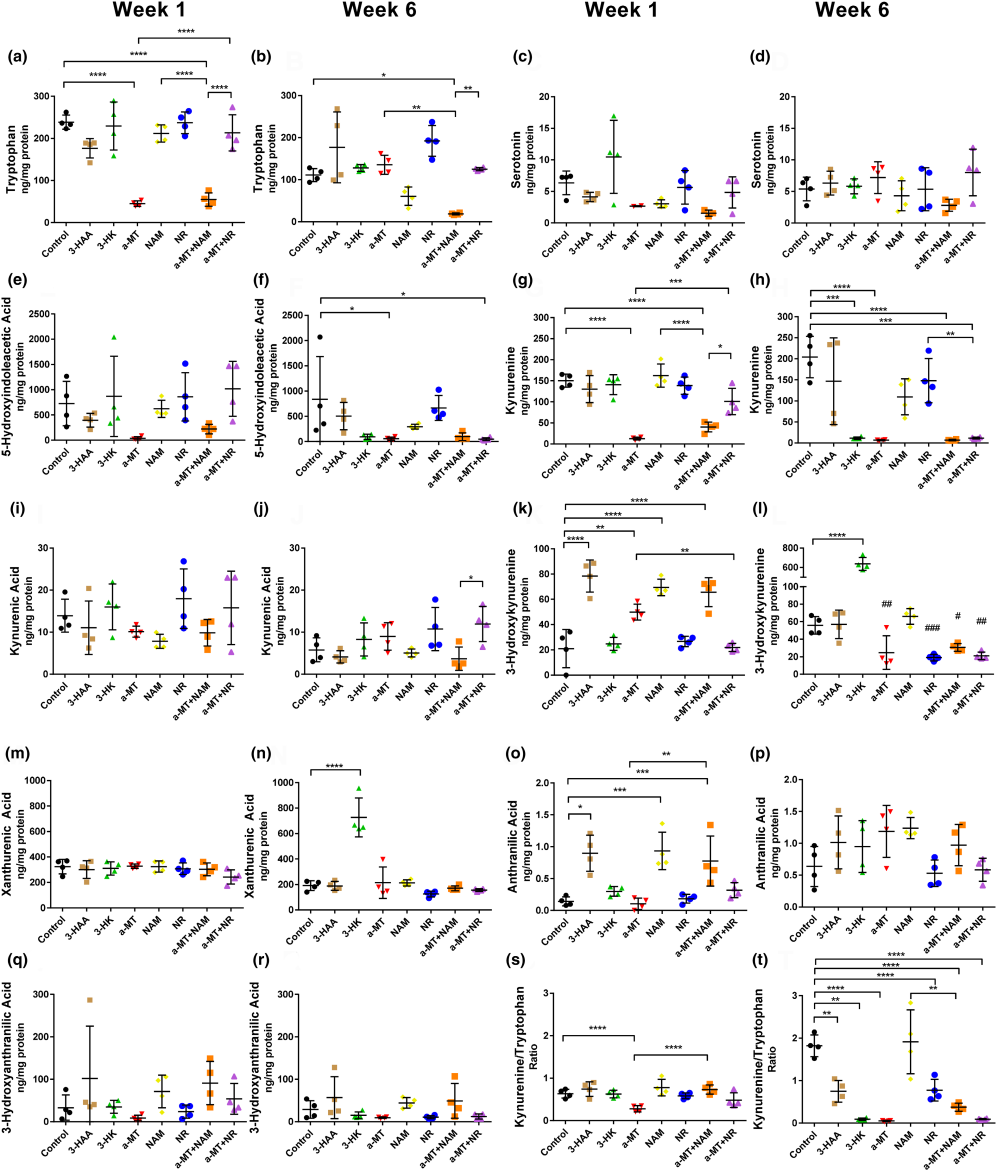
Systemic levels of kynurenines and other tryptophan‐related metabolites in DGRP_229 *Drosophila* after kynurenine pathway manipulation. Systemic levels of tryptophan, serotonin pathway, and kynurenine pathway metabolites were measured in flies after 1 week and 6 weeks on their respective diets (*n* = 4 samples per treatment group with each sample representing seven to eight flies). We used a targeted metabolomic approach to measure tryptophan (a, b), serotonin (c, d), 5‐hydroxyindoleacetic acid (e, f), kynurenine (g, h), kynurenic acid (i, j), 3‐hydroxykynurenine (k, l), xanthurenic acid (m, n), anthranilic acid (o, p), 3‐hydroxyanthranilic acid (q, r), and the kynurenine to tryptophan ratio (s, t). Data were analyzed using one‐way ANOVA with Tukey's multiple comparisons test. *p < 0.05, **p < 0.01, ***p < 0.001, ****p < 0.0001. (l) # indicates metabolites were significantly altered when compared to the control using one‐way ANOVA with Dunnett's multiple comparisons test after excluding the group which was exogenously fed 3‐HK. #*p* < 0.05, ##*p* < 0.01, ###*p* < 0.001.

We then measured the effects of the KP manipulation strategies on systemic levels of tryptophan, serotonin pathway, and kynurenine pathway metabolites at 1 week and 6 weeks on each respective diet (Figure 7). Metabolites were extracted from whole frozen flies and normalized to the amount of protein in each sample. Tryptophan was reduced in both α‐MT and α‐MT + NAM fed flies compared to the control at Week 1; however, it was only reduced in α‐MT + NAM fed flies by Week 6 (Figure 7a,b). Serotonin was not significantly altered by any treatment (Figure 7c,d), and 5‐hydroxyindolacetic acid was reduced in α‐MT, and α‐MT + NR fed flies at Week 6 (Figure 7e,f). Kynurenine levels were reduced in flies fed α‐MT and α‐MT + NAM compared to controls at Week 1 and in flies fed α‐MT, α‐MT + NAM, and α‐MT + NR compared to controls by Week 6 (Figure 7g,h).

Interestingly, flies fed 3‐HK also had reduced kynurenine levels compared to controls at Week 6 (Figure 7h). Kynurenic acid levels were not significantly altered by any of the diets compared to controls (Figure 7i,j). 3‐hydroxykynurenine was elevated in flies fed 3‐HAA, α‐MT, NAM, and α‐MT + NAM compared to controls after 1 week on each diet (Figure 7k). After 6 weeks, systemic 3‐hydroxykynurenine levels were over 10‐fold higher in flies fed 3‐hydroxykynurenine (3‐HK) than in controls (Figure 7l). When flies fed exogenous 3‐HK were excluded from the analysis, α‐MT, NR, α‐MT + NAM, and α‐MT + NR all had significantly reduced levels of 3‐hydroxykynurenine after 6 weeks on their respective diets (Figure 7l). Xanthurenic acid was elevated in flies fed 3‐HK after 6 weeks on the diet (Figure 7m,n). Anthranilic acid was elevated in flies fed 3‐HAA, NAM, and α‐MT + NAM after 1 week on each diet (Figure 7o); however, these effects did not persist up to Week 6 (Figure 7p). Unexpectedly, 3‐hydroxyanthranilic acid was not significantly altered by any diets (Figure 7q,r). We also measured α‐methyltryptophan and saw it was significantly elevated in flies fed α‐MT and α‐MT + NAM after 1 week on each diet (Figure S4A), and it was significantly elevated in flies fed α‐MT, α‐MT + NAM, and α‐MT + NR after 6 weeks on their respective diets (Figure S4B).

### 2.7 ^13^C_11_, ^15^N_2_‐tryptophan tracer study

To further understand tryptophan metabolism in *Drosophila*, we used stable isotope resolved metabolomics on flies fed labeled ^13^C_11_, ^15^N_2_‐tryptophan and traced the metabolic journey of the labeled isotopologues through the tryptophan‐kynurenine pathway. We showed that xanthommatin, cardinalic acid, and xanthurenic acid are abundantly synthesized from tryptophan while small amounts of partially labeled NAD^+^ and nicotinamide mononucleotide were present. Our results highlight the dynamic and complex nature of this biochemical process as illustrated in (Figures S6–S8).

### 2.8 Kynurenine pathway manipulation alters expression of pathway‐ and longevity‐associate genes associated with longevity

**FIGURE 8:**
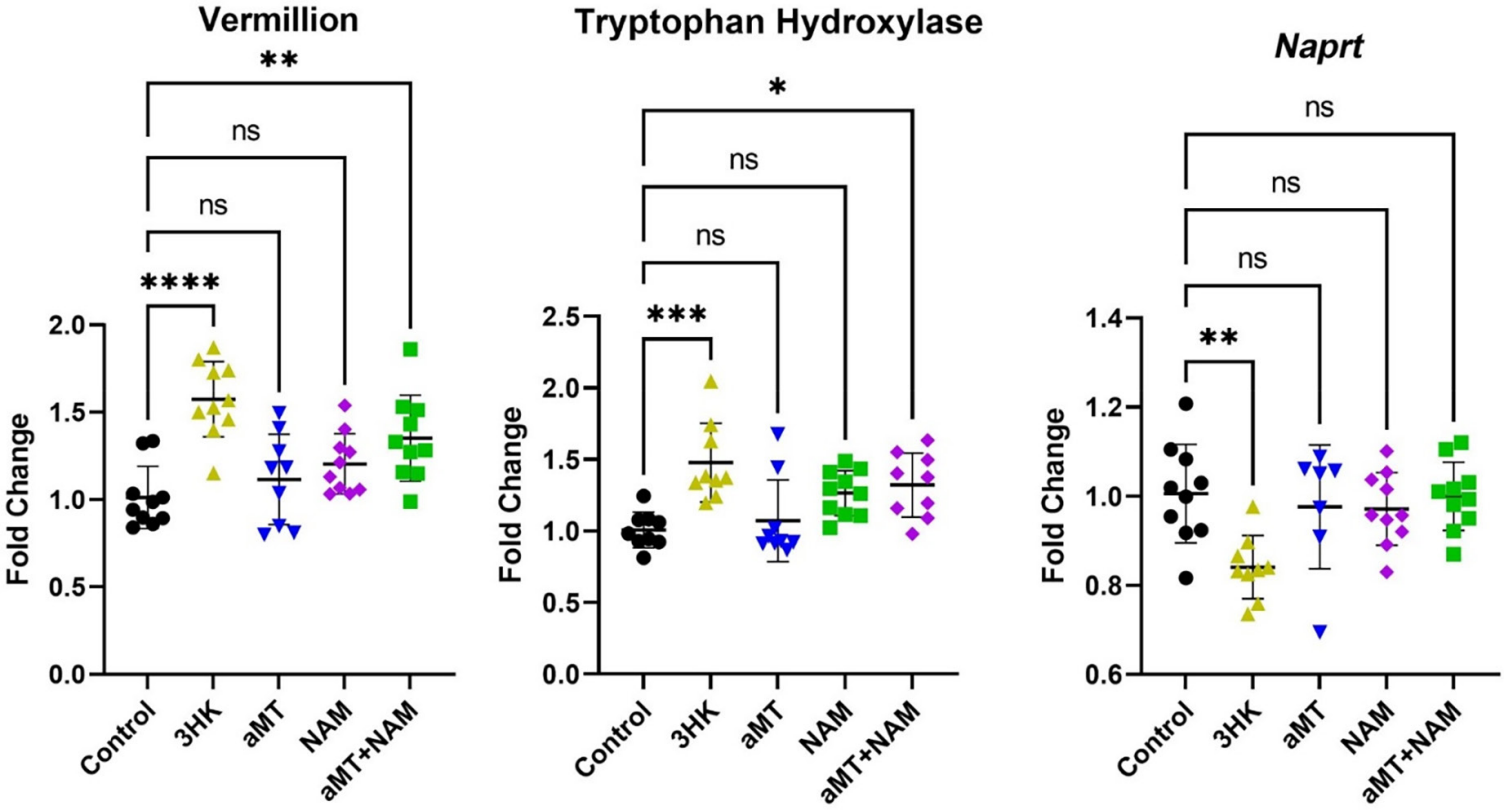
Gene expression of KP enzymes in *Drosophila* in response to 1 month treatment with either 3‐HK, α‐MT, NAM, or α‐MT + NAM. Fold change analysis of genes in the kynurenine pathway and NAD^+^ biosynthetic pathway (*n* = 7–10 per treatment group). Vermilion (tryptophan 2,3‐dioxygenase) catalyzes the conversion of L‐tryptophan to N‐formyl‐L‐kynurenine. Tryptophan hydroxylase catalyzes the hydroxylation of tryptophan which is the initial and rate‐limiting step in the synthesis of the neurotransmitter serotonin. *Naprt* catalyzes the conversion of nicotinic acid to nicotinic acid mononucleotide. Data were analyzed using one‐way ANOVA with Tukey's multiple comparisons test. *p < 0.05, **p < 0.01, ***p < 0.001, ****p < 0.0001.

We evaluated the expression of the KP genes, NAD^+^ biosynthetic pathway and key longevity genes following 1 month treatment with either 3‐HK, α‐MT, NAM, or α‐MT + NAM. Our findings provide insight into how the treatments influence gene expression and may consequently impact survivorship and life span. For genes in the KP and NAD^+^ biosynthetic pathway, we found that the expression of *Naprt* significantly decreased upon treatment with 3‐HK (Figure 8). This suggests a potential inhibitory effect of 3‐HK on *Naprt* expression highlighting the interaction between these two pathways. Moreover, treatment with 3‐HK resulted in an upregulation of *Tryptophan hydroxylase* and *vermilion*. Interestingly, the dual treatment of α‐MT + NAM also led to an increase in the expression of *Tryptophan hydroxylase* and *vermilion* (Figure 8). KP genes with nonsignificant changes resulting from the treatments can be found in Figure S9. These results underscore the complex regulatory interactions between these genes and the metabolic pathways mediated by the treatments.

**FIGURE 9:**
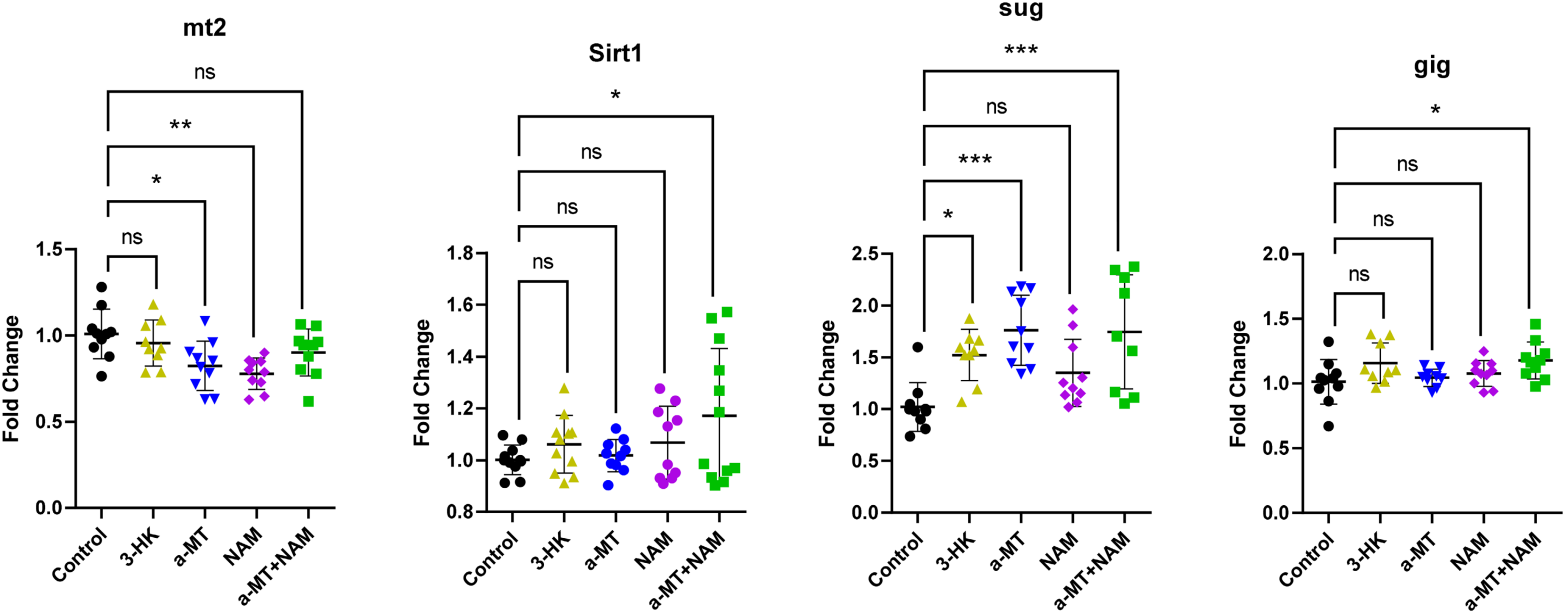
Expression of longevity‐associated genes in *Drosophila* in response to 1 month treatment with either 3‐HK, α‐MT, NAM, or α‐MT + NAM. Fold change analysis of genes associated with longevity in *Drosophila* (*n* = 10 per treatment group). Data were analyzed using one‐way ANOVA with Tukey's multiple comparisons test. *p < 0.05, **p < 0.01, ***p < 0.001, ****p < 0.0001.

In the analysis of longevity‐associated genes, we found that the expression of Mt2 significantly decreased after treatment with α‐MT and NAM, however, this downregulation was not observed in 3‐HK or dual treatment groups (Figure 9). SUG gene expression was increased with 3‐HK, α‐MT, and α‐MT + NAM treatment. SIRT1, and gig showed a significant increase in expression only in flies treated with α‐MT + NAM treatment, indicating that activation of these genes may be a mechanism of the extended longevity resulting from this treatment (Figure 9). Longevity genes with nonsignificant changes can be found in Figure S10.

Collectively, these results suggest a complex network of interactions between the KP and NAD^+^ biosynthetic pathways, influenced by the applied treatments. The data also suggests that dual treatment with α‐MT + NAM, could positively modulate the expression of key longevity genes and contribute to increased life span and survival.

## 3 DISCUSSION

Our findings (summarized in Figure S5) show that manipulating specific components of the kynurenine pathway and NAD^+^ salvage pathway through dietary intervention alters life span and the capacity for physical function in *Drosophila melanogaster*. Importantly, we provide proof‐of‐concept evidence that KP suppression in combination with NAD^+^ precursor supplementation synergistically increases life span and significantly preserves physical function. This study also, for the first time, examines changes in systemic levels of tryptophan, serotonin pathway, and kynurenine pathway metabolites resulting from aging and from manipulating KP with dietary interventions in *Drosophila*.

We identified metabolite diets that led to decreased physical performance compared to flies on control diets (3‐HAA and 3‐HK), diets that did not have significant effects on physical activity (NAM), diets that provided modest benefit in preserving physical function at older ages (α‐MT and NR) and the combination diets (α‐MT + NAM and α‐MT + NR) which significantly preserved physical function at an older age in every category we measured. Though these diets were administered from eclosure, the beneficial effects of the combination treatments were only apparent after 5 weeks, indicating that they significantly delayed the onset of functional decline in older flies. Interestingly, the deficits in physical function elicited by 3‐HK occurred after 1 week, while the negative effects from 3‐HAA were not significant until Week 5, indicating that 3‐HK may have been more immediately detrimental while 3‐HAA required a cumulative effect to cause significant changes.

Findings from our dendrite anatomical analyses are consistent with the effects of the dietary treatments on the physical performance assays and may indicate that combination treatments beneficially support dendrite metabolism and growth. This may be a key mechanism through which KP modulation elicits benefits that manifest as preserved physical performance at an older age. Notably, flies fed 3‐HK had a numeric but not significant decrease in dendritic density. This trend is in agreement with studies that have shown that 3‐HK is neurotoxic in *Drosophila* (Campesan et al., 2011) and mammals (Okuda et al., 1998; Ramírez‐Ortega et al., 2017; Westbrook et al., 2020). Future studies to measure the effects of these diets on other cell types such as glial cells as well as other organs are warranted. Recent clinical studies have demonstrated the benefits of NAD^+^ supplementation to neurological conditions. NR supplementation has been shown to improve coordination and eye movement in patients with ataxia telangiectasia (Presterud et al., 2023), increase cerebral NAD^+^ levels associated with altered brain metabolism and clinical improvement (Brakedal et al., 2022), and stimulate mitophagy which reverses memory impairment in nematodes, mice, and human cells (Fang, Hou, Palikaras, et al., 2019). These studies highlight the potential for NAD^+^ supplementation as a therapeutic intervention and hint at the possibility for additional benefits from dual KP suppression and NAD^+^ supplementation in the treatment of neurodegenerative diseases.

KP manipulation induced changes in survivorship that mirrored changes seen in physical performance. Dietary supplementation with 3‐HK and 3‐HAA both led to significantly decreased average life span compared to control flies. α‐MT, NAM, and NR significantly increased average life span, and NAM additionally increased maximum life span compared to controls. These findings agree with Oxenkrug et al. (2011), who showed that α‐MT could extend average but not maximum life span in *Drosophila*. These findings are also in agreement with studies of mutant *vermilion* flies that have decreased tryptophan conversion to kynurenine and live longer (Oxenkrug, 2010). Our findings with diets containing NAM and NR are novel to our knowledge, and they are in agreement with findings that these NAD^+^ precursors can extend life span in *C. elegans* (Mouchiroud et al., 2013). Interestingly, flies consuming diets containing combinations of α‐MT + NAM or α‐MT + NR had significantly increased average and maximum life span compared to flies consuming the control diet or any of the other singular component diets. The gains in maximum life spans of combination diet‐fed flies were beyond the additive effect of the gains of flies fed any of the singular component diets demonstrating that a synergistic benefit is attainable through suppressing KP flux while supplementing NAD^+^ levels.

Clues as to the role of the KP in aging come from the comparisons of systemic levels of tryptophan, serotonin pathway, and kynurenine pathway metabolites in young flies at 1 week to older flies at 6 weeks of age. Compared to young flies, aged flies had decreased tryptophan, kynurenic acid, and xanthurenic acid and increased 3‐HK, anthranilic acid, and an increased kynurenine‐to‐tryptophan ratio. These findings are largely in agreement with numerous studies, including our own, that show that aging and frailty are accompanied by decreased systemic levels of tryptophan, elevated kynurenine‐to‐tryptophan ratio, and elevated 3‐HK (Cervenka et al., 2017; Westbrook et al., 2020).

Findings from the metabolomic measurement of systemic tryptophan, serotonin pathway, and KP metabolite levels resulting from KP manipulation diets provide insight into factors that may contribute to the effects of these diets. Diets that led to shortened life span all had significantly higher 3‐HK at a point in their life span. This decrease in life span is validated in studies of *cardinal* mutant *Drosophila* which have elevated levels of 3‐HK and also have shortened life spans and accelerated age‐related declines (Savvateeva et al., 2000). The flies that were fed 3‐HK had a 10‐fold elevation in systemic levels of 3‐HK and elevated xanthurenic acid, formed from 3‐HK, after 6 weeks on this diet. Flies fed 3‐HAA had elevated 3‐HK and anthranilic acid only in Week 1. Curiously, we saw only small nonsignificant elevations in 3‐HAA in flies that were fed this metabolite, and additionally, there were no age‐related changes in the levels of this metabolite. In our labeled tryptophan tracer study, labeled 3‐HAA was not found. This indicates that the majority of the 3‐HAA fed to the flies may have been rapidly converted to other metabolites, including 3‐HK. This leaves open the possibility that the detrimental effects of the 3‐HAA diet could be mediated by excess production of 3‐HK. While the impacts of the 3‐HAA diet on life span and physical performance were clear, the full contributions of this diet to the changes in KP metabolism are unclear from the metabolites we were able to measure in this study.

Evidence that both 3‐HAA and 3‐HK can be cytotoxic, neurotoxic and generate oxidative stress (Eastman & Guilarte, 1989; Goldstein et al., 2000; Okuda et al., 1998; Ramírez‐Ortega et al., 2017) provides a potential mechanism for the effects of these metabolites on life span. Contrastingly, some reports show that 3‐HAA is a neuro‐protective in vitro (Leipnitz et al., 2007) and in vivo (Krause et al., 2011). Similarly, studies show that 3‐HAA is cardio‐protective in some mouse models (Zhang et al., 2012) while others show 3‐HAA as causal in some cardiovascular diseases (Wang et al., 2017). These seemingly conflicting observations vary in species studied, cells or tissue used, dosage used, duration of treatment and other experimental conditions making comparisons difficult however some observations can in part be explained by the dual nature of the metabolites. Depending on the physiological state of the cell, 3‐HK and 3‐HAA can be free radical scavengers or toxic (Ramírez‐Ortega et al., 2017). Further, this toxicity was shown to be independent of ROS, indicating other mechanisms may be at play. The effects of chronic administration of these metabolites on life span and health span of *Drosophila* have not been explored elsewhere to our knowledge.

Inflammatory cytokines are stimuli for the production and subsequent accumulation of downstream kynurenines such as 3‐HK with age in mammals (Cervenka et al., 2017; Schwarcz et al., 2012; Westbrook et al., 2020); however, this connection is not well understood in fruit flies. *Drosophila* experience elevated inflammation with age (Kounatidis et al., 2017; Nainu et al., 2019), and studies have shown that the *TDO2* gene, which encodes tryptophan 2,3 dioxygenase—which begins the conversion of tryptophan to kynurenine—is upregulated upon sensory neuronal tumor necrosis factor (*TNF*/*Eiger* in *Drosophila*) overexpression (Jo et al., 2017) and is upregulated with aging (Landis et al., 2012). Future studies are needed to determine the effects of inflammation on KP metabolism in *Drosophila*.

Flies fed NAM or NR had few changes in systemic levels of tryptophan, serotonin pathway, and KP metabolites. Flies fed NR had significantly decreased systemic levels of 3‐HK at Week 6. Flies fed NAM had elevated systemic levels of 3‐HK and anthranilic acid compared to controls at Week 1, indicating that the NAM diet may have transiently activated KP metabolism. A similar effect was seen in studies in mice where chronic NAM supplementation improved health span measures in mice without extending life span (Mitchell et al., 2018). In this study, NAM supplementation caused tryptophan depletion, elevation of IDO expression, and presumably, higher levels of kynurenines, although this was not measured. This unexpected result suggests an unknown feedback mechanism whereby supplementing a precursor of NAD^+^ led to activation of KP flux. Studies investigating the effects of suppressing KP metabolism in mammals are warranted. Dual KP suppression and NAD^+^ supplementation may be a particularly effective in reducing the effects of age in mammals because the KP is necessary for de novo NAD^+^ synthesis (Bender, 1983; Canto et al., 2015) and blocking KP metabolism alone could reduce NAD^+^ availability.

All flies fed α‐MT had dramatically lowered kynurenine levels confirming that the metabolite does effectively inhibit TDO in flies (Lancaster & Sourkes, 1969), preventing the conversion of tryptophan into kynurenine to the degree that kynurenine was barely detectable by Week 6. All groups of flies fed diets containing α‐MT had significantly reduced 3‐HK as well, indicating that KP metabolites downstream of kynurenine were affected by TDO inhibition. Future studies will be needed to determine if this affects NAD^+^ levels. The α‐MT + NAM and α‐MT + NR combination diets caused multiple, sometimes differing, effects on systemic levels of tryptophan, serotonin pathway, and KP metabolites. However, decreased levels of kynurenine and 3‐HK were observed at old age in flies fed both diets. Flies fed the combination of a‐MT + NAM had significantly reduced 3‐HK levels compared to flies fed NAM in Week 6 even though flies fed NAM had similar levels of 3‐HK compared to controls. This may indicate that lowered 3‐HK is the factor that contributes to the better effect of the combination. In fact, four of the five dietary interventions that led to increased life span caused decreases in systemic levels of 3‐HK. Considering the significant elevations in 3‐HK levels with age, the pro‐aging effects of exogenously elevating this metabolite, and the reduction of age‐related effects via its suppression, the action of 3‐HK may play an important role in aging in *Drosophila*. NAD^+^ precursors improve mitochondrial function in *C. elegans* (Mouchiroud et al., 2013) and may work through similar mechanisms in flies. It is plausible that the benefits of the combination diets are attributable to decreased toxicity from 3‐HK and improved mitochondrial function.

Supplementation with α‐MT + NAM increased *SIRT1* and *gig* expression. Both genes are activated in response to caloric restriction (Marygold & Leevers, 2002; Rogina & Helfand, 2004) and their overexpression is associated with increased life span in *Drosophila* (Kapahi et al., 2004; Whitaker et al., 2013). These findings indicate that increased sirtuin activity and decreased TOR signaling may mediate the effects of this diet on life span. The expression of *Naprt* which converts nicotinic acid to nicotinic acid mononucleotide, was decreased only in flies fed a diet supplemented with 3‐HK highlighting another indirect interaction between the KP and NAD^+^ biosynthetic pathways in *Drosophila*.

Tryptophan and its metabolites are important biochemical signaling molecules, chemical precursors, and coenzymes involved in a number of processes in *Drosophila*. While the pathway from tryptophan to NAD^+^ is well described in mammals, the connections between these metabolites are less clear in *Drosophila*. The fruit fly lacks orthologs for both 3‐hydroxyanthranilic acid dioxygenase, which converts 3‐HAA to quinolinic acid in mammals, and quinolinate phosphoribosyltransferase (QPRTase), which converts quinolinic acid to nicotinic acid mononucleotide in mammals. Accordingly, we were not able to detect quinolinic acid in any flies in this study. Instead, some of the major downstream products of tryptophan were xanthurenic acid, xanthommatin, and cardinalic acid. Interestingly, we saw small amounts of partially labeled NAD^+^ in flies fed ^13^C_11_, ^15^N_2_‐tryptophan for 6 days. The isotopologues of NAD^+^ found in this study (M + 1, M + 2, and M + 3) differ from those generated during NAD^+^ biosynthesis in mammals (Liu et al., 2018) (M + 6) and may be the result of multiple rounds of forward and reverse reactions. The microbiome could also contribute to these results as both prokaryotes and eukaryotes have direct tryptophan to NAD^+^ biosynthetic pathways (Kurnasov et al., 2003).

There were some limitations to this study. We used metabolites we thought most impactful for exploring the effects of KP and NAD^+^ biosynthetic pathway manipulation in this study. We focused on the effects of downstream kynurenines; however, using kynurenine or kynurenic acid as a dietary supplement may provide insights that improve our understanding of the role of this pathway in aging. While we have investigated metabolites related to tryptophan in this study, future studies will examine the changes occurring in the wider metabolome including metabolites related to energy metabolism and biosynthetic pathways. We were unable to measure NAD^+^ related metabolites in our samples, leaving exploration of the impacts of these interventions on levels of NAD^+^ related metabolites for future studies. We used only one concentration of each dietary KP metabolite leaving open the possibility of greater success being attainable by altering these concentrations. While α‐MT is a TDO inhibitor in insects (Lancaster & Sourkes, 1969), it does not inhibit TDO in mammals (Moran & Sourkes, 1963). At the protein level, TDO *vermilion* is 56% identical and 72% similar to human TDO2 (DIOPT V9.0) (Huang et al., 2013; Searles et al., 1990). Fortunately, there are numerous IDO and TDO inhibitors that can be utilized in mammals (Breda et al., 2016; Peng et al., 2022). In this study, we treated flies from eclosure throughout their life span as a proof‐of‐concept study to determine if these treatments affect life span and health span. We must leave it to future studies to determine whether late‐stage supplementation of α‐MT, α‐MT + NAM, and α‐MT + NR can extend life span. In depth studies of the relationship between KP and NAD+ modulation and mitochondrial dynamics, muscle fiber composition, muscle function and physical performance are also warranted in future studies.

In summary, we show for the first time that pharmacologically inhibiting the KP while supplementing with precursors of NAD^+^ provides robust preservation of physical function, dramatically increases life span, and supports dendritic structure. We also show that chronically elevating downstream KP metabolites induced decreased physical function, shortened life span, and diminished dendritic structure. We show that normal aging leads to key changes in systemic levels of KP metabolites, including decreased tryptophan and increased kynurenine to tryptophan ratio and 3‐HK. This work highlights the potential for pharmacologically manipulating these pathways to develop practical interventions which reduce age‐related decline in physical function.

## 4 MATERIALS AND METHODS

### 4.1 DGRP_229 stock and maintenance

Virgin males of DGRP_229 from the *Drosophila* Genetic Reference Panel (Mackay et al., 2012) were used for all assays. All stocks and control groups were fed standard food medium (solid ingredients: 79% cornmeal, 16% yeast, and 5% agar). Treated groups were fed a standard food medium infused with a specific metabolite found in the kynurenine pathway. The flies were maintained in vials, grouped by 30 flies per vial. Food was replaced every 2 days. Flies were stored in a warm room at 25°C with 55% humidity.

### 4.2 DGRP_229 drug treatments

There were five metabolites tested: 3‐Hydroxyanthranilic acid (3‐HAA), 3‐hydroxykynurenine (3‐HK), α‐methyl‐D, L‐tryptophan (α‐MT), NAM, and NR. The metabolites were tested separately and in combination, as follows: α‐methyl‐D, L‐tryptophan + NAM and α‐methyl‐D, L‐tryptophan + NR. Figure 1 displays the KP pathway areas targeted in this study. To model elevations in potentially deleterious kynurenines, we fed flies 3‐HK and 3‐HAA (Figure 1a). To examine the effects of KP blockade or NAD^+^ precursor supplementation, we fed flies either α‐MT, NAM, or NR (Figure 1b). To test whether administering KP blockade in tandem with NAD^+^ precursor supplementation elicited any additional benefit, we fed flies combinations of α‐MT + NAM or α‐MT + NR (Figure 1c).

The concentration of each metabolite in fly food fed to treated flies was 1 mg/mL of 3‐HAA, 1 mg/mL of 3‐HK^15^, 34.5 mM (7.53 mg/mL) of α‐methyl‐D, L‐tryptophan (Oxenkrug et al., 2011), 0.2 mg/mL of NAM (Jia et al., 2008), and 0.2 mg/mL of NR. The concentrations of the treatments were chosen based on previous studies reporting dose–response curves, lethal doses, and/or optimal doses showing a physiological effect. The concentrations of 3‐HAA and NR were matched with those of 3‐HK and NAM respectively. Flies were treated chronically upon eclosure. Food was replenished every other day. The raw metabolites were stored at −20°C, and food was stored at 4°C.

### 4.3 Feeding rate assay

We used the Capillary Feeder Assay (Ja et al., 2007) to account for potential differences among treatments in feeding rates on the physical performance measures. Groups of four 5‐day‐old males from each treatment were placed in a vial and allowed to feed on either 1‐mM sucrose or 1 mg/mL of 3‐HAA, 1 mg/mL of 3‐HK, 34.5 mM (1.6 × 10^−4^ mg/mL) of α‐methyl‐D, L‐tryptophan, 0.2 mg/mL of NAD^+^, or 0.2 mg/mL of NR with sucrose from capillary tubes (#53432–706, VWR) for 24 h. To account for the effect of evaporation, we calculated mean evaporation from control 1‐mM sucrose (*n* = 10) and sucrose plus treatment (*n* = 10) capillaries using vials without flies. Food loss by evaporation or consumption by flies was measured using a digital caliper. We used the following formula to determine total consumption: food consumption of flies (μL) = (food loss [μL] − evaporative loss [μL])/total mg of flies in the vial.

### 4.4 Physical performance assay

We assessed the physical performance by measuring the speed and endurance of individual flies (Gabrawy et al., 2019). We used 30 virgin male flies per treatment at each age: 1 week (young age), 3 weeks (middle age), five (middle‐old age), and 7 weeks (old age). Briefly, each fly was aspirated into a serological pipette and tapped down to the bottom. Once the fly body passed the zero cm mark during the upwards climb, the trial began, and the timer started. Once the fly reached the 9 cm mark, the time was noted, and the climbing speed was calculated. Although endurance was assessed in a similar capacity, it was based on the distance the fly reached in 15 s with a maximum distance of 27 cm.

### 4.5 Survivorship assay

We measured the life span of the flies by using eight Plexiglas population cages (20 L × 21w × 21.5 h cm), with one population treated with control food and seven populations treated with standard fly food infused with a kynurenine metabolite, as described in Gabrawy et al. 2019. DGRP_229 male flies were placed in population cages upon eclosure, 250 flies per cage. Twenty milliliters of control or treated food was placed in 100 × 15 mm BD Falcon plastic Petri dishes and were replaced every other day during mortality data collection. Mortality data were collected by counting and removing the dead flies every other day. We tested eight different treatments: control (standard fly food medium) and standard fly food infused with the following metabolites: 3‐HAA, 3‐HK, α‐methyl‐D, L‐tryptophan, NAM, NR, α‐methyl‐D, L‐tryptophan + NAD^+^ and α‐methyl‐D, L‐tryptophan + NR. Survivorship data were analyzed by Cox regression proportional hazards models (PROC PHREG, SAS V9.3). We used reduced models to compare survivorship of control and metabolite‐treated flies for each genotype separately. We also used a full model in which we compared the effects of genotype and treatment in a single model.

### 4.6 Reverse transcriptase‐PCR (RT‐PCR)

For RT‐PCR measurements virgin male DGRP_229 flies were treated with their respective diets (control, 3HK, NAM, α‐MT, and α‐MT + NAM) for 31 days post eclosure. Flies were then frozen on dry ice and stored at −80°C.

cDNA was synthesized from 300 ng of total RNA using High‐Capacity cDNA Reverse Transcription Kits (# 4368813, Thermo Fisher Scientific) according to the instructions of the manufacturer. A volume of 2 μL of cDNA was PCR amplified using PowerTrack SYBR Green in a 20 μL reaction volume. Reaction mixtures were incubated in a QuantStudio 6 Pro system using the default thermal cycling parameters & ramp rates.

The fold change calculations were carried out using the Cq mean values of the replicate groups. Only samples with replicate group Cq confidence scores <0.8, Cq standard deviation of >0.5 were included in the analysis. The data were entered into Prism Graphpad software using ordinary one‐way ANOVA and analyzed by comparing the mean of each column to the mean of the control column. The fold changes were then plotted against the treatment groups on a scatterplot displaying means with SD. Primer selection and info are provided in the supplemental section below. We used SYBR Green Primers to study key genes in this pathway, including those for Tryptophan hydroxylase, Formamidase, Kynurenine aminotransferase, *vermilion*, and nonfunctional mutant *cinnabar* (CN) from the strain C1. We also studied the expression of *rp49*, as it serves as a housekeeping gene, providing a reference point for the relative quantification of target gene expression.

On the other hand, genes associated with longevity in *Drosophila*, including *Foxo*, *Sirt2*, *Indy*, *Naam*, *chico*, *sug*, *HDAC1*, *Mt2*, *inaE*, *Sirt1*, *gig*, and *RP49* were studied using TaqMan Probes. These genes are known to play a significant role in life span determination and health span (Komatsu et al., 2019).

### 4.7 Metabolomic measurements

#### 4.7.1 Internal standard synthesis

Stock solutions of each analyte of interest (5 ng/μL each) were made in DI water and stored at −80°C. To prepare internal standards, stock solutions were derivatized in a comparable manner to samples using isotopically labeled benzoyl chloride (^13^C_6_‐BZC) as follows: 200 μL of the stock solution was mixed with 400 μL each of 500 mM NaCO_3_ (aq), and 2% ^13^C_6_‐BZC in acetonitrile was added to the solution. After 2 min, the reaction was stopped by adding 400 μL of 20% acetonitrile in water containing 3% sulfuric acid. The solution was mixed well and stored in 10 μL aliquots at −80°C. One aliquot was diluted 100x with 20% acetonitrile in water containing 3% sulfuric acid to make the working internal standard solution used in the sample analysis.

#### 4.7.2 Extraction

Flies were homogenized using an ultrasonic dismembrator in 100–750 μL of 0.1 M TCA, which contained 10^−2^ M sodium acetate, 10^−4^ M EDTA, and 10.5% methanol (pH 3.8). Ten microliters of homogenate were used for protein quantification. Samples were spun in a microcentrifuge at 10,000 g for 20 min at 4°C. The supernatant was removed for LC/MS analysis.

#### 4.7.3 Benzoyl chloride derivatization and LC/MS analysis

Analytes in tissue extract supernatant were quantified using liquid chromatography/mass spectrometry (LC/MS) following derivatization with benzoyl chloride (BZC). Five microliters of supernatant was then mixed with 10 μL each of 500 mM NaCO_3_ (aq) and 2% BZC in acetonitrile in an LC/MS vial. After 2 min, the reaction was stopped by adding a 10 μL internal standard solution.

LC was performed on a 2.1 × 100 mm, 1.6 μm particle CORTECS Phenyl column (Waters Corporation, Milford, MA, USA) using a Waters Acquity UPLC. Mobile phase A was 0.1% aqueous formic acid, and mobile phase B was acetonitrile with 0.1% formic acid. MS analysis was performed using a Waters Xevo TQ‐XS triple quadrupole tandem mass spectrometer. The source temperature was 150°C, and the desolvation temperature was 400°C. The LC gradient is shown in Table S2. Metabolite measurements were completed in the Vanderbilt University Neurochemistry Core.

### 4.8 Stable isotope‐resolved metabolomics

One‐week‐old male DGRP_229 flies were placed on holidic media (Piper et al., 2014) with ^13^C_11_, ^15^N_2_‐tryptophan for 6 days then snap frozen. The frozen flies were homogenized in 80% massspectrometry grade methanol for metabolite extraction. The samples were then centrifuged to collect the supernatant which contained metabolites. For data acquisition, a liquid chromatography‐mass spectrometer system was employed. Specifically, the Thermo Scientific Mass Spectrometer was coupled with a Vanquish UHPLC system. Throughout the data acquisition process, the autosampler chamber was carefully maintained at 4°C to ensure the integrity of the metabolite samples. Reverse phase chromatographic separation was carried out using a Discovery HSF5 column (Sigma) with a Supelco guard column, both operating at a constant temperature of 35°C. The applied method involved mobile phases: (A) 0.1% formic acid in MS‐water and (B) 0.1% formic acid in acetonitrile. The run time for the entire method was 15 min for each sample. Full MS scans, as well as full MS/ddMS2 (top10) scans, were obtained in the initial 11‐minute window, which was followed by a 4‐minute period for column re‐equilibration. To ensure the sensitivity and data accuracy of the system, routine calibration procedures were performed prior to starting data acquisition. The acquired data underwent analysis using Thermo Fisher FreeStyle and TraceFinder software for identification of metabolites and quantification of all isotopologues of identified metabolites, respectively. Specifically, identification was achieved through fragmentation pattern and m/z accuracy matching, while quantification was achieved through integrating the chromatographic peaks to extract intensity. The obtained intensities were subsequently normalized based on the protein concentration of the respective sample. For calculating fold change, the intensity data for each isotopologue was divided by the average of the M + 0 isotopologue.

### 4.9 Protein assay

Protein concentration was determined using the BCA Protein Assay Kit (Thermo Scientific, Waltham, MA, USA) in a 96‐well plate format. Ten microliters of tissue homogenate was mixed with 200 μL of mixed BCA reagent per manufacturer instructions. The plate was incubated at 23°C for 2 h before absorbance was measured by the plate reader (POLARstar Omega), purchased from BMG LABTECH Company.

### 4.10 Dendrite density

Virgin males of fly stock C380 were collected and treated chronically upon eclosure. The flies were maintained in vials, grouped by 30 flies a vial. All stocks and control groups were fed standard food medium (solid ingredients: 79% cornmeal, 16% yeast, and 5% agar), and food was replenished every other day. Flies were stored in a warm room at 25°C with 55% humidity under a 12‐h light and dark cycle.

To analyze dendritic anatomy, we used the well‐characterized flight motoneurons located on adult flies' VNC. There are five singly identifiable motoneurons on each side of the VNC that innervate the dorsal longitudinal muscle involved in wing depression (Consoulas et al., 2002; Ikeda & Koenig, 1988). The developmental processes and functional properties of these motoneurons, particularly MN5, have been characterized in great detail (Heinrich & Ryglewski, 2020; Ryglewski et al., 2017; Vonhoff et al., 2013; Vonhoff & Duch, 2010) and have served as a model to study the anatomical effects of genetic manipulations affecting molecules involved in neurological diseases (Hutchinson et al., 2014; Mishra‐Gorur et al., 2014; Vonhoff et al., 2012). Flight motoneurons were labeled via the C380‐GAL4, UAS‐mCD8‐GFP; *cha*‐GAL80 driver that expresses GAL4 predominately in motoneurons and a few other unidentified neurons in the adult VNC (Sanyal, 2009; Vonhoff et al., 2013).

Adults were dissected in PBS, fixed in 4% paraformaldehyde, and mounted in glycerol. Optical sections of bilateral motoneuron dendrites were acquired with a 63x oil‐immersion lens on a Leica SP5 confocal laser scanning microscope located in the Keith R. Porter Imaging Facility, University of Maryland, Baltimore County. Image acquisition settings were standardized to 16‐bit, 1024 × 1024‐pixel resolution, 2x line averaging, and 1 μm step size. Laser power and gain settings were adjusted for each age‐related data set, using control samples dendritic fluorescence to optimize dynamic range. All samples for each age‐related data set were imaged in one session using the same imaging parameters.

The dissection followed Boerner & Godenschwege (2011) methodology. Briefly, flies were anesthetized via CO_2_ and placed on a petri dish. Under high magnification, the legs, proboscis, and wings were removed, with insect pins used to elongate the fly. Submerged in *Drosophila* saline, a shallow incision was made from the abdomen to the thorax midline. With the thorax held open by two insect pins, the internal organs were removed, exposing the dorsal‐ventral nerve and cervical connective. The motoneuron was exposed as described in Ryglewski et al. (2017). Motoneuron dendritic arborizations were defined as the region of interest, and GFP fluorescence was measured as the mean gray value within that region. Background fluorescence was subtracted, as described in Mishra‐Gorur et al. (2014).

Relative fluorescence intensity was compared among each age‐related data set. Image analysis was performed using Fiji/ImageJ software (NIH). For each Z‐stack, maximum intensity projections were created, and the free‐hand selection tool was used to mark the boundaries of dendritic fluorescence. Mean gray values were obtained for each dendritic ROI and subtracted from the mean gray value of background fluorescence acquired from a selected area outside the ROI for each image. The average from all mean gray values was calculated for each treatment and normalized to the age‐related control. The difference between treatment and control was calculated to estimate the net effect of each treatment. Negative values represent lower fluorescent levels corresponding to fewer dendrites as compared to controls, whereas positive values correspond to higher fluorescent levels and an increased number of dendrites as compared to controls. Average mean gray values were normally distributed (Shapiro–Wilk test; *p* > 0.05) and compared with one‐way ANOVA and Dunnett's post hoc tests using SPSS Statistics 27 (IBM).

### 4.11 Statistics and reproducibility

Climbing speed is defined as the time taken to reach the short distance of 9 cm, and endurance is defined as the distance reached in 15 s; data were analyzed with ANOVA and Dunn's multiple comparison test. Failure rate data were measured as the percentage of flies unable to complete the short‐distance climbing speed and/or long‐distance endurance assays due to stopping or falling. Failure rate data were analyzed with Fisher's exact test. Survivorship data were measured as duration until the death event. Data were analyzed by Cox regression proportional hazards models (PROC PHREG, SAS V9.3). We used reduced models to compare survivorship of control and metabolite‐treated flies for each genotype separately. We also used a full model in which we compared the effects of genotype and treatment in a single model. Maximal life span was calculated as the mean age of the oldest 10% of the population within that group, and significance was determined using ANOVA and Dunn's multiple comparison test. Dendrite density was measured in terms of relative fluorescence intensity and was compared among each age‐related data set. Average mean gray values were normally distributed (Shapiro–Wilk test; *p* > 0.05) and compared with one‐way ANOVA and Dunnett's post hoc tests using SPSS Statistics 27 (IBM). Gene expression data were analyzed using one‐way ANOVA with Tukey's multiple comparisons test. Feeding rate data were analyzed with ANOVA and Dunnett's multiple comparison test. We completed the climbing assay (*n* = 30 per group) and the longevity assay (*n* = 250 per group) for flies treated with a‐MT, 3‐HAA, NAM, and NAM + a‐MT twice.

## AUTHOR CONTRIBUTIONS

RW, MG, and PA conceived and designed the study. RW contributed the kynurenine and NAD^+^ pathway manipulation strategies. RW and MG developed the metabolite diets for *Drosophila*. MG, AK, NK, NO, and RW managed fly stocks for the experiment. MG, AK, NK, and NO performed in vivo experiments and survivorship assays. FV and TD performed histology. GM performed metabolomic measurements. RW, MG, and FV performed statistical analyses. JF, RW, MG, and PA designed the graphical abstract. Most authors were responsible for the final version's critical manuscript revision and approval. PA and JW supervised the work.

## CONFLICT OF INTEREST STATEMENT

The authors declare that there are no conflicts of interest regarding the publication of this paper. This includes, but is not limited to, financial, personal, or professional affiliations that could be perceived to influence the content or interpretation of the findings within this manuscript.

## Supporting information


Appendix S1:

Appendix S2:

## Data Availability

The data that support the findings of this study are available from the corresponding author upon reasonable request.
