# Supplementary material for: Dual treatment with kynurenine pathway inhibitors and NAD + precursors synergistically extends life span in Drosophila
Source: Aging Cell. 2024 Mar 13;23(4):e14102. doi: 10.1111/acel.14102 (PMC11019140; doi:10.1111/acel.14102)
Supplement: Supplementary file 1 — Appendix S1: [file ACEL-23-e14102-s002.pdf]

Detailed Materials and Methods

DGRP_229 Stock and Maintenance

Virgin males of DGRP_229 from the *Drosophila* Genetic Reference Panel[54] were used for all assays. All stocks and control groups were fed standard food medium (solid ingredients: 79% cornmeal, 16% yeast, and 5% agar). Treated groups were fed a standard food medium infused with a specific metabolite found in the kynurenine pathway. The flies were maintained in vials, grouped by thirty flies per vial. Food was replaced every two days. Flies were stored in a warm room at 25ºC with 55% humidity.

DGRP_229 Drug Treatments

There were five metabolites tested: 3-Hydroxyanthranilic acid (3-HAA), 3-hydroxykynurenine (3-HK), α-methyl-D, L-tryptophan (α-MT), nicotinamide (NAM), and nicotinamide riboside (NR). The metabolites were tested separately and in combination, as follows: α-methyl-D, L-tryptophan + NAM and α-methyl-D, L-tryptophan + NR. Figure 1 displays the KP pathway areas targeted in this study. To model elevations in potentially deleterious kynurenines, we fed flies 3-HK and 3-HAA (**fig 1, column A)**. To examine the effects of KP blockade or NAD+ precursor supplementation, we fed flies either α-MT, NAM, or NR **(fig 1, column B).** To test whether administering KP blockade in tandem with NAD+ precursor supplementation elicited any additional benefit, we fed flies combinations of α-MT+NAM or α-MT+NR **(fig 1, column C)**.

The concentration of each metabolite in fly food fed to treated flies was 1 mg/ml of 3-HAA, 1 mg/ml of 3-HK[15], 34.5 mM (7.53 mg/ml) of α-methyl-D, L-tryptophan[25], 0.2 mg/ml of NAM[55], and 0.2 mg/ml of NR. The concentrations of the treatments were chosen based on previous studies reporting dose-response curves, lethal doses, and/or optimal doses showing a physiological effect. The concentrations of 3-HAA and NR were matched with those of 3-HK and NAM respectively. Flies were treated chronically upon eclosure. Food was replenished every other day. The raw metabolites were stored at -20ºC, and food was stored at 4ºC.

Feeding Rate Assay

We used the Capillary Feeder Assay[30] to account for potential differences among treatments in feeding rates on the physical performance measures. Groups of four 5-day-old males from each treatment were placed in a vial and allowed to feed on either 1-mM sucrose or 1 mg/ml of 3-HAA, 1 mg/ml of 3-HK, 34.5 mM (1.6×10^{-4} mg/ml) of α-methyl-D, L-tryptophan, 0.2 mg/ml of NAD, or 0.2 mg/ml of NR with sucrose from capillary tubes (#53432–706, VWR) for 24 hours. To account for the effect of evaporation, we calculated mean evaporation from control 1-mM sucrose (*n* = 10) and

sucrose plus treatment (*n* = 10) capillaries using vials without flies. Food loss by evaporation or consumption by flies was measured using a digital caliper. We used the following formula to determine total consumption: food consumption of flies (µl) = (food loss [µl] − evaporative loss [µl])/total mg of flies in the vial.

Physical Performance Assay

We assessed the physical performance by measuring the speed and endurance of individual flies[56]. We used 30 virgin male flies per treatment at each age: one week (young age), three weeks (middle age), five (middle-old age), and seven weeks (old age). Briefly, each fly was aspirated into a serological pipette and tapped down to the bottom. Once the fly body passed the zero cm mark during the upwards climb, the trial began, and the timer started. Once the fly reached the 9 cm mark, the time was noted, and the climbing speed was calculated. Although endurance was assessed in a similar capacity, it was based on the distance the fly reached in 15 seconds with a maximum distance of 27cm.

Survivorship Assay

We measured the lifespan of the flies by using eight Plexiglas population cages (20*l* × 21*w* × 21.5*h* cm), with one population treated with control food and seven populations treated with standard fly food infused with a kynurenine metabolite, as described in Gabrawy *et al.* 2019. DGRP_229 male flies were placed in population cages upon eclosure, 250 flies per cage. Twenty milliliters of control or treated food were placed in 100 × 15 mm BD Falcon plastic Petri dishes and were replaced every other day during mortality data collection. Mortality data were collected by counting and removing the dead flies every other day. We tested eight different treatments: control (standard fly food medium) and standard fly food infused with the following metabolites: 3-HAA, 3-HK, α-methyl-D, L-tryptophan, NAM, NR, α-methyl-D, L-tryptophan + NAD and α-methyl-D, L-tryptophan + NR. Survivorship data were analyzed by Cox regression proportional hazards models (PROC PHREG, SAS V9.3). We used reduced models to compare survivorship of control and metabolite-treated flies for each genotype separately. We also used a full model in which we compared the effects of genotype and treatment in a single model.

Reverse transcriptase-PCR (RT-PCR)

For RT-PCR measurements virgin male DGRP_229 flies were treated with their respective diets (control, 3HK, NAM, α-MT, and α-MT+NAM) for 31 days post eclosure. Flies were then frozen on dry ice and stored at -80°C.

cDNA was synthesized from 300ng of total RNA using High-Capacity cDNA Reverse Transcription Kits (# 4368813, Thermo Fisher Scientific) according to the instructions of the manufacturer. A volume of 2ul of cDNA was PCR amplified using PowerTrack SYBR Green in a 20ul reaction volume. Reaction mixtures were incubated in a QuantStudio 6 Pro system using the default thermal cycling parameters & ramp rates.

The fold change calculations were carried out using the Cq mean values of the replicate groups. Only samples with replicate group Cq confidence scores greater than 0.8, Cq standard deviation of less than 0.5 were included in the analysis. The data was entered into Prism Graphpad software using ordinary one-way ANOVA and analyzed by comparing the mean of each column to the mean of the control column. The fold changes were then plotted against the treatment groups on a scatterplot displaying means with SD. Primer selection and info are provided in the supplemental section below. We used SYBR Green Primers to study key genes in this pathway, including those for Tryptophan hydroxylase, Formamidase, Kynurenine aminotransferase, Vermilion, and nonfunctional mutant cinnabar (CN) from the strain C1. We also studied the expression of *rp49*, as it serves as a housekeeping gene, providing a reference point for the relative quantification of target gene expression.
On the other hand, genes associated with longevity in *Drosophila*, including *Foxo*, *Sirt2*, *Indy*, *Naam*, *chico*, *sug*, *HDAC1*, *Mt2*, *inaE*, *Sirt1*, *gig*, and *RP49* were studied using TaqMan Probes. These genes are known to play a significant role in lifespan determination and healthspan[57].

Metabolomic Measurements

Internal Standard Synthesis
Stock solutions of each analyte of interest (5ng/µl each) were made in DI water and stored at -80°C. To prepare internal standards, stock solutions were derivatized in a comparable manner to samples using isotopically labeled benzoyl chloride ($^{13}C_6$-BZC) as follows: 200 µl of the stock solution was mixed with 400 µl each of 500 mM $NaCO_3$ (aq), and 2% $^{13}C_6$-BZC in acetonitrile was added to the solution. After two minutes, the reaction was stopped by adding 400 µl of 20% acetonitrile in water containing 3% sulfuric acid. The solution was mixed well and stored in 10 µl aliquots at -80°C. One aliquot was diluted 100x with 20% acetonitrile in water containing 3% sulfuric acid to make the working internal standard solution used in the sample analysis.

Extraction
Flies were homogenized using an ultrasonic dismembrator in 100-750 µl of 0.1M TCA, which contained 10^{-2} M sodium acetate, 10^{-4} M EDTA, and 10.5 % methanol (pH 3.8). Ten microliters of homogenate were used for protein quantification. Samples were spun

in a microcentrifuge at 10,000 g for 20 minutes at 4°C. The supernatant was removed for LC/MS analysis.

Benzoyl Chloride Derivatization and LC/MS Analysis
Analytes in tissue extract supernatant were quantified using liquid chromatography/mass spectrometry (LC/MS) following derivatization with benzoyl chloride (BZC). Five µl of supernatant was then mixed with 10 µl each of 500 mM $NaCO_3$ (aq) and 2% BZC in acetonitrile in an LC/MS vial. After two minutes, the reaction was stopped by adding a 10 µl internal standard solution.

LC was performed on a 2.1 x 100 mm, 1.6 µm particle CORTECS Phenyl column (Waters Corporation, Milford, MA, USA) using a Waters Acquity UPLC. Mobile phase A was 0.1% aqueous formic acid, and mobile phase B was acetonitrile with 0.1% formic acid. MS analysis was performed using a Waters Xevo TQ-XS triple quadrupole tandem mass spectrometer. The source temperature was 150°C, and the desolvation temperature was 400°C. The LC gradient is shown in supplemental Table 2. Metabolite measurements were completed in the Vanderbilt University Neurochemistry Core.

Stable Isotope Resolved Metabolomics

One week-old male DGRP_229 flies were placed on holidic media[58] with $^{13}C_{11}$, $^{15}N_2$-tryptophan for 6 days then snap frozen. The frozen flies were homogenized in 80% mass-spectrometry (MS) grade methanol for metabolite extraction. The samples were then centrifuged to collect the supernatant which contained metabolites. For data acquisition, a liquid chromatography-mass spectrometer system was employed. Specifically, the Thermo Scientific Mass Spectrometer was coupled with a Vanquish UHPLC system. Throughout the data acquisition process, the autosampler chamber was carefully maintained at 4°C to ensure the integrity of the metabolite samples. Reverse phase chromatographic separation was carried out using a Discovery HSF5 column (Sigma) with a Supelco guard column, both operating at a constant temperature of 35°C. The applied method involved mobile phases: (A) 0.1% formic acid in MS-water and (B) 0.1% formic acid in acetonitrile. The run time for the entire method was 15 minutes for each sample. Full MS scans, as well as full MS/ddMS2 (top10) scans, were obtained in the initial 11-minute window, which was followed by a 4-minute period for column re-equilibration. To ensure the sensitivity and data accuracy of the system, routine calibration procedures were performed prior to starting data acquisition. The acquired data underwent analysis using Thermo Fisher FreeStyle and TraceFinder software for identification of metabolites and quantification of all isotopologues of identified metabolites, respectively. Specifically, identification was achieved through fragmentation pattern and m/z accuracy matching, while quantification was achieved through integrating the chromatographic peaks to extract intensity. The obtained intensities were subsequently normalized based on the protein concentration of the respective sample. For calculating fold change, the intensity data for each isotopologue was divided by the average of the M+0 isotopologue.

Protein Assay

Protein concentration was determined using the BCA Protein Assay Kit (Thermo Scientific, Waltham, MA, USA) in a 96-well plate format. Ten μl of tissue homogenate was mixed with 200 μl of mixed BCA reagent per manufacturer instructions. The plate was incubated at 23°C for two hours before absorbance was measured by the plate reader (POLARstar Omega), purchased from BMG LABTECH Company.

Dendrite Density

Virgin males of fly stock C380 were collected and treated chronically upon eclosure. The flies were maintained in vials, grouped by 30 flies a vial. All stocks and control groups were fed standard food medium (solid ingredients: 79% cornmeal, 16% yeast, and 5% agar), and food was replenished every other day. Flies were stored in a warm room at 25°C with 55% humidity under a 12-hour light and dark cycle.

To analyze dendritic anatomy, we used the well-characterized flight motoneurons located on adult flies' ventral nerve cords (VNC). There are five singly identifiable motoneurons on each side of the ventral nerve cord that innervate the dorsal longitudinal muscle involved in wing depression[59,60]. The developmental processes and functional properties of these motoneurons, particularly MN5, have been characterized in great detail[61-64] and have served as a model to study the anatomical effects of genetic manipulations affecting molecules involved in neurological diseases[31,65,66]. Flight motoneurons were labeled via the C380-GAL4, UAS-mCD8-GFP; cha-GAL80 driver that expresses GAL4 predominately in motoneurons and a few other unidentified neurons in the adult VNC[62,67].

Adults were dissected in PBS, fixed in 4% paraformaldehyde, and mounted in glycerol. Optical sections of bilateral motoneuron dendrites were acquired with a 63x oil-immersion lens on a Leica SP5 confocal laser scanning microscope located in the Keith R. Porter Imaging Facility, University of Maryland, Baltimore County. Image acquisition settings were standardized to 16-bit, 1024 × 1024-pixel resolution, 2x line averaging, and 1 μm step size. Laser power and gain settings were adjusted for each age-related data set, using control samples dendritic fluorescence to optimize dynamic range. All samples for each age-related data set were imaged in one session using the same imaging parameters.

The dissection followed Boerner *et al*. (2011) methodology[68]. Briefly, flies were anesthetized via CO_2 and placed on a petri dish. Under high magnification, the legs, proboscis, and wings were removed, with insect pins used to elongate the fly. Submerged in *Drosophila* saline, a shallow incision was made from the abdomen to the thorax midline. With the thorax held open by two insect pins, the internal organs were removed, exposing the dorsal-ventral nerve and cervical connective. The motoneuron

was exposed as described in Ryglewski *et al*. (2016). Motoneuron dendritic arborizations were defined as the region of interest, and GFP fluorescence was measured as the mean gray value within that region. Background fluorescence was subtracted, as described in Mishra-Gorur *et al.* (2014)[31].

Relative fluorescence intensity was compared among each age-related data set. Image analysis was performed using Fiji/ImageJ software (NIH). For each Z-stack, maximum intensity projections were created, and the free-hand selection tool was used to mark the boundaries of dendritic fluorescence. Mean gray values were obtained for each dendritic ROI and subtracted from the mean gray value of background fluorescence acquired from a selected area outside the ROI for each image. The average from all mean gray values was calculated for each treatment and normalized to the age-related control. The difference between treatment and control was calculated to estimate the net effect of each treatment. Negative values represent lower fluorescent levels corresponding to fewer dendrites as compared to controls, whereas positive values correspond to higher fluorescent levels and an increased number of dendrites as compared to controls. Average mean gray values were normally distributed (Shapiro-Wilk test; $P > 0.05$) and compared with one-way ANOVA and Dunnett's post hoc tests using SPSS Statistics 27 (IBM).

Statistics and Reproducibility

Climbing speed is defined as the time taken to reach the short distance of 9 cm, and endurance is defined as the distance reached in 15 seconds; data were analyzed with ANOVA and Dunn's multiple comparison test. Failure rate data were measured as the percentage of flies unable to complete the short-distance climbing speed and/or long-distance endurance assays due to stopping or falling. Failure rate data were analyzed with the Fisher's Exact Test. Survivorship data were measured as duration until the death event. Data were analyzed by Cox regression proportional hazards models (PROC PHREG, SAS V9.3). We used reduced models to compare survivorship of control and metabolite-treated flies for each genotype separately. We also used a full model in which we compared the effects of genotype and treatment in a single model. Maximal life span was calculated as the mean age of the oldest 10% of the population within that group, and significance was determined using ANOVA and Dunn's multiple comparison test. Dendrite density was measured in terms of relative fluorescence intensity and was compared among each age-related data set. Average mean gray values were normally distributed (Shapiro-Wilk test; $P > 0.05$) and compared with one-way ANOVA and Dunnett's post hoc tests using SPSS Statistics 27 (IBM). Gene expression data were analyzed using 1-way ANOVA with Tukey's multiple comparisons test. Feeding rate data were analyzed with ANOVA and Dunnett's multiple comparison test.
