# Supplementary material for: Dual treatment with kynurenine pathway inhibitors and NAD + precursors synergistically extends life span in Drosophila
Source: Aging Cell. 2024 Mar 13;23(4):e14102. doi: 10.1111/acel.14102 (PMC11019140; doi:10.1111/acel.14102)
Supplement: Supplementary file 2 — Appendix S2: [file ACEL-23-e14102-s001.docx]

**Supplemental Tables & Figures**

**Supplemental Figure 1. The effects of aging on physical performance of DGRP_229 flies.** Climbing speed **(A)**, Endurance **(B)**, and their respective failure rates (**C** and **D**) were measured in flies receiving no treatments at 1 week, 3 weeks, 5 weeks, and 7 weeks of age. Significance was determined using the Kruskal-Wallis 1-way ANOVA with Dunn’s multiple comparisons test (**A** and **B**) and with Fisher's Exact Test (**C** and D). **P* < 0.05, ***P* < 0.01, ****P* < 0.001, *****P* < 0.0001

| **Table 1A. Survivorship descriptive statistics** | | |
| --- | --- | --- |
| **Log-rank (Mantel-Cox) test** | | **P- value** |
| **Control vs** | **3-HAA** | < 0.0001 |
| **Control vs** | **3-HK** | < 0.0001 |
| **Control vs** | **α-MT** | < 0.0001 |
| **Control vs** | **NAM** | < 0.0001 |
| **Control vs** | **NR** | < 0.0001 |
| **Control vs** | **α-MT + NAM** | < 0.0001 |
| **Control vs** | **α-MT + NR** | < 0.0001 |
| **Combinations compared to singular components** | | |
| **α-MT vs** | **α-MT + NAM** | < 0.0001 |
| **α-MT vs** | **α-MT + NR** | 0.0013 |
| **NAM vs** | **α-MT + NAM** | < 0.0001 |
| **NR vs** | **α-MT + NR** | < 0.0001 |

| **Table 1. Mean duration (days)** | | | | | | | | | |
| --- | --- | --- | --- | --- | --- | --- | --- | --- | --- |
|  | **Control** | **3-HAA** | **3-HK** | **α-MT** | **NAM** | **NR** | **α-MT + NAM** | **α-MT + NR** |  |
| Mean (SE) |  | 43.84 | 42.88 | 60.71 | 58.71 | 57.54 | 71.72 | 64.47 |  |
| **Percent difference in mean duration** | | | | | | | | | |
|  | **Control** | **3-HAA** | **3-HK** | **α-MT** | **NAM** | **NR** | **α-MT + NAM** | **α-MT + NR** |  |
| **Control** | 0 | -14.78 | -17.35 | 17.11 | 14.29 | 12.55 | 29.83 | 21.95 |  |
| **3-HAA** | 14.78 | 0 | -2.23 | 27.79 | 25.33 | 23.81 | 38.87 | 32 |  |
| **3-HK** | 17.35 | 2.23 | 0 | 29.37 | 26.96 | 25.48 | 40.21 | 33.49 |  |
| **α-MT** | -17.11 | -27.79 | -29.37 | 0 | -3.41 | -5.51 | 15.35 | 5.83 |  |
| **NAM** | -14.29 | -25.33 | -26.96 | 3.41 | 0 | -2.03 | 18.14 | 8.93 |  |
| **NR** | -12.55 | -23.81 | -25.48 | 5.51 | 2.03 | 0 | 19.77 | 10.75 |  |
| **α-MT + NAM** | -29.83 | -38.87 | -40.21 | -15.35 | -18.14 | -19.77 | 0 | -11.25 |  |
| **α-MT + NR** | -21.95 | -32 | -33.49 | -5.83 | -8.93 | -10.75 | 11.25 | 0 |  |

**Supplemental Table 1.** Descriptive statistics of the effects of treatment on survivorship (**A**) The effects of treatment on the mean duration of DGRP_229 flies and the percent difference in the mean duration between treatments (**B**).

**Supplemental Figure 2.** The addition of metabolites had no significant effect on the feeding rate. The feeding rate (uL/mg) was measured using the CAFE assay.

**Supplemental Figure 3**. The effects of treatments on maximum lifespan. Maximum lifespan was estimated by the mean age of death of the oldest 10% in each group. Data were analyzed with Kruskal-Wallis 1-way ANOVA with Dunn’s multiple comparisons test. **P* < 0.05, ***P* < 0.01, ****P* < 0.001, *****P* < 0.0001.

**Supplemental Figure 4**. Systemic levels of α-methyltryptophan were measured in flies after one week (A) and six weeks (B) on their respective diets (n=4 samples per group, with each sample representing 7- 8 flies).


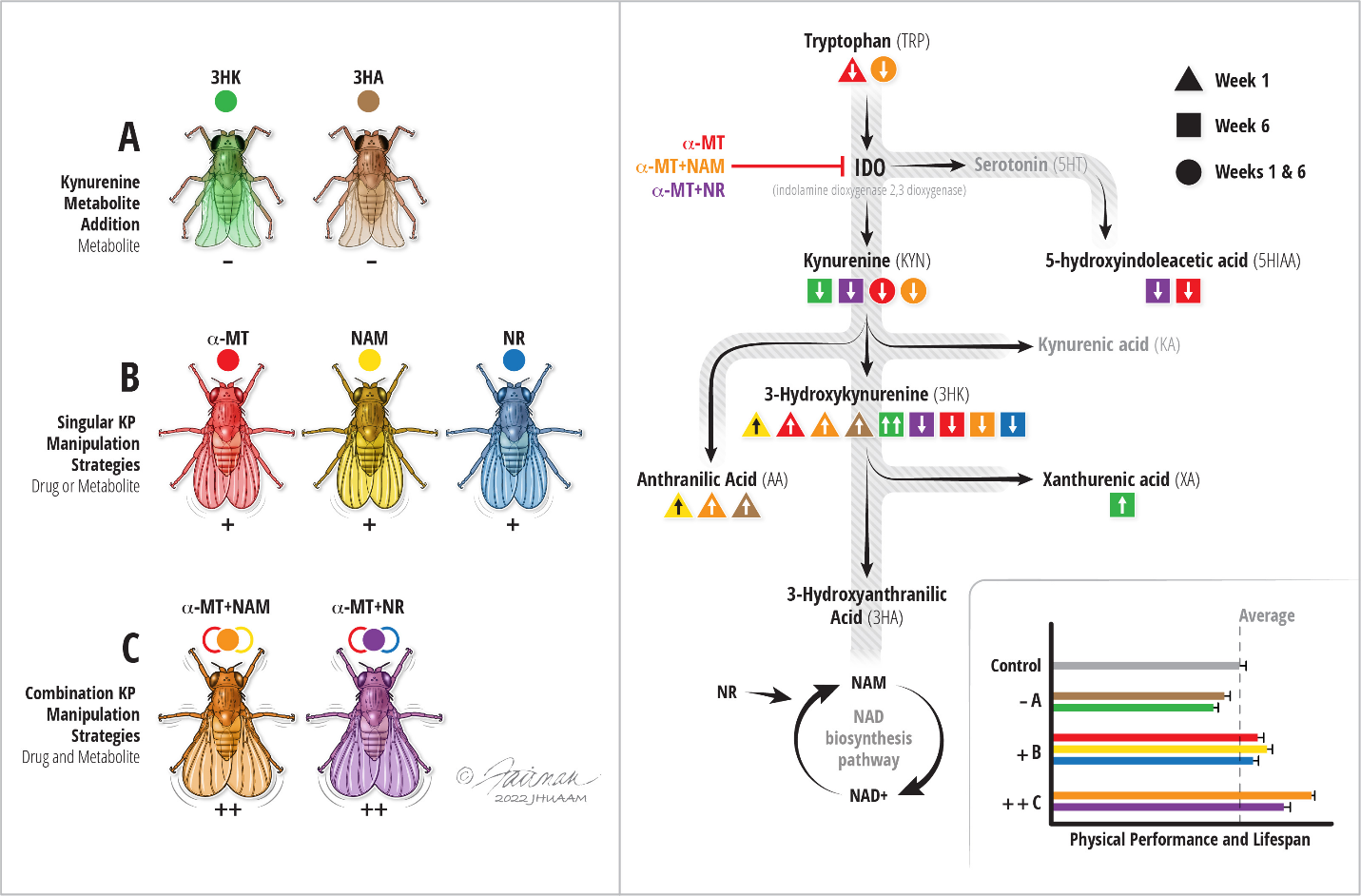


**Supplemental Figure 5.** Graphical abstract summarizing the changes in systemic levels of tryptophan, serotonin pathway, and kynurenine pathway metabolites and physical performance and lifespan in response to kynurenine pathway manipulation treatments. Flies fed 3-HK and 3-HAA **(A)**. Flies fed α-MT, NAM, or NR **(B).** Flies fed combinations of α-MT+NAM or α-MT+NR **(C)**. Treatment groups are color-coded in the schematic pathway map and physical performance and lifespan plot. Significant changes in metabolites in week 1 have a triangle symbol, changes in week 6 have a square symbol and changes in both weeks have a circle symbol.

|  | | | | |
| --- | --- | --- | --- | --- |
| **Time** | **Flow rate** | **%A** | **%B** | **Gradient** |
| 0.00 | 0.200 | 99 | 1 | -- |
| 0.10 | 0.200 | 93 | 7 | 6 |
| 0.50 | 0.200 | 85 | 15 | 6 |
| 14.0 | 0.200 | 45 | 55 | 6 |
| 14.5 | 0.200 | 30 | 70 | 6 |
| 18.0 | 0.200 | 1 | 99 | 6 |
| 19.0 | 0.200 | 1 | 99 | 6 |
| 19.1 | 0.200 | 99 | 1 | 6 |
| 22.0 | 0.200 | 99 | 1 | 6 |

**Supplemental Table 2.** HPLC gradient for tryptophan, serotonin pathway, and kynurenine pathway metabolite analysis.

**
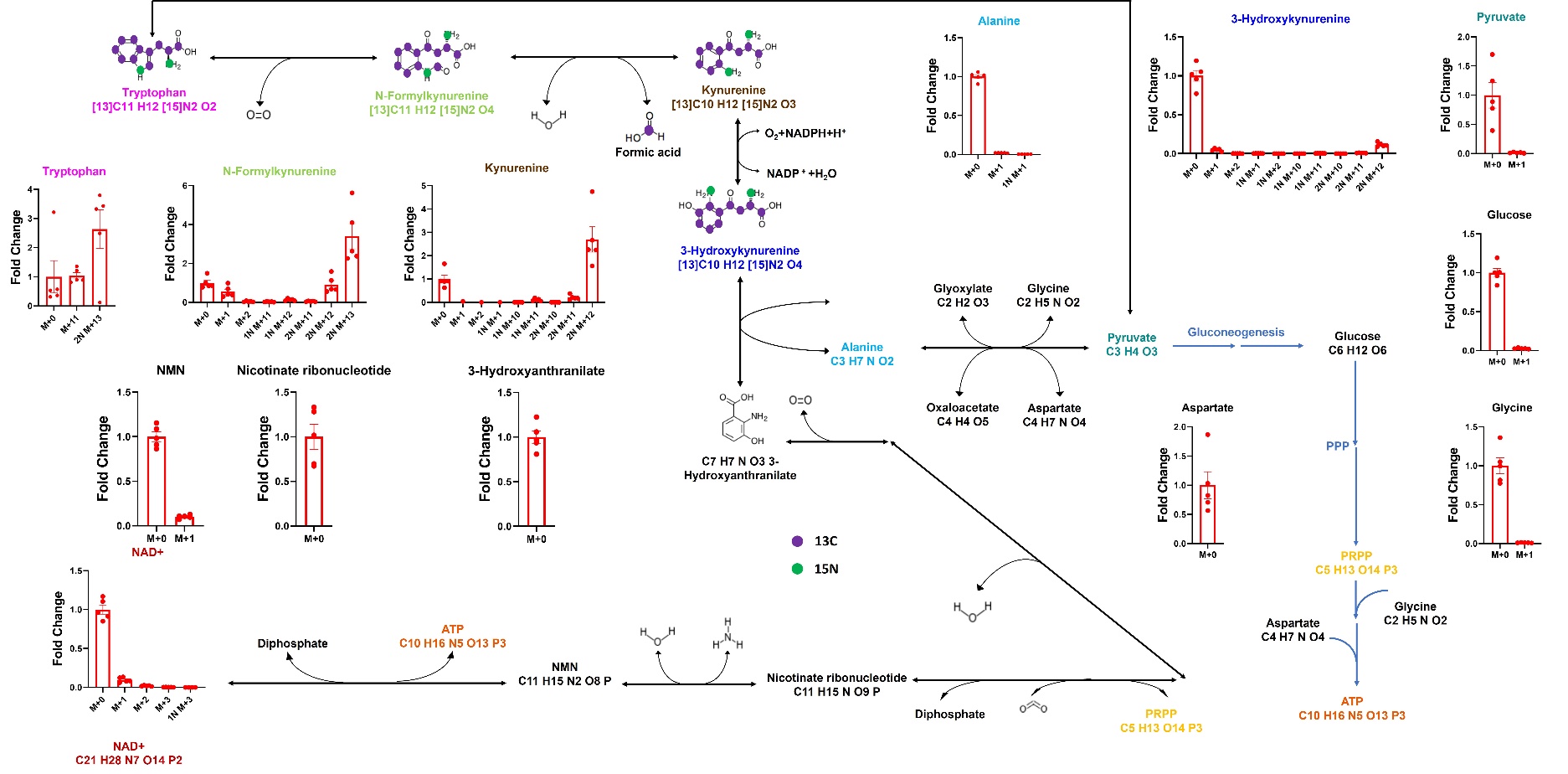
**

**Supplemental Figure 6:** **Tryptophan contributes to NAD^+^ production.**

Fold change for all detected isotopologues of each metabolite within the tryptophan-kynurenine-NAD^+^ pathway with respect to their m+0 isotopologue and the corresponding pathway illustration is shown. Purple dots represent labeled ^13^C and green dots represent labeled ^15^N. Data are shown as mean fold change ± SEM. Stable isotope resolved metabolomics was performed on the flies grown in ^13^C_11_, ^15^N_2_-tryptophan. As expected, we detected fully labeled isotopologue of tryptophan (m+13 or mass of the parent ions ^13^C_11_, ^15^N_2_-tryptophan equal to the mass [m] of ^12^C_11_, ^14^N_2_-tryptophan plus 13 more mass units due to ^13^C_11_, ^15^N_2_-tryptophan, Figure 6 pink) present in the flies grown in ^13^C_11_, ^15^N_2_-tryptophan at 2.6 times higher than the non-labeled M+0 tryptophan. The next 3 products of fully labeled m+13 tryptophan, including fully labeled m+13 isotopologue of N-formylkynurenine (Figure 6 green), m+12 isotopologue of kynurenine (Figure 6 brown), and m+12 isotopologue of 3-hydroxykyrenine (Figure 6 dark blue) were also detected. These results indicate that the flies did uptake and utilize the ^13^C_11_, ^15^N_2_-tryptophan through the tryptophan-kynurenine pathway. Besides the fully labeled isotopologues, several additional isotopologues of these metabolites were detected, indicating multiple rounds of forward and reverse reactions.

Interestingly, both labeled ^13^C and ^15^N were also detected in NAD^+^ (Figure 6, red), with the highest labeled isotopologues observed being m+3. This m+3 NAD^+^ was detected with either three carbon labeled as ^13^C or two carbon labeled as ^13^C and one nitrogen labeled as ^15^N. The m+3 isotopologues can be explained by the conversion of 3-hydroxykyrenine to alanine (Figure 6, light blue). Alanine, in turn, is converted to pyruvate (Figure 6, blueish green), which can produce glucose through gluconeogenesis. Alternatively, tryptophan can convert directly to pyruvate. Glucose through the pentose phosphate pathway (PPP) contributes to phosphoribosyl diphosphate (PRPP) (Figure 6 yellow), which can then produce ATP (Figure 6, orange) via purine metabolism. Both PRPP and ATP can obtain labeled ^13^C through alanine or pyruvate. ATP can also obtain labeled ^15^N from glycine and aspartate, both of which can become labeled via transamination reaction couple to the conversion of alanine to pyruvate. Because ^13^C_11_, ^15^N_2_-tryptophan is the only source of ^13^C and ^15^N in flies, the presence of labeled ^13^C and ^15^N in NAD^+^ after subtracting natural abundance demonstrates that tryptophan contributed to the production of NAD^+^.

Of note, we detected non-labeled isotopologues of 3-hydroxyanthranilate, nicotinate ribonucleotide, NMN, alanine, pyruvate, glucose, and glycine, but not the non-endogenous labeled isotopologues. This could be due to the fast conversion of these labeled isotopologues.

**
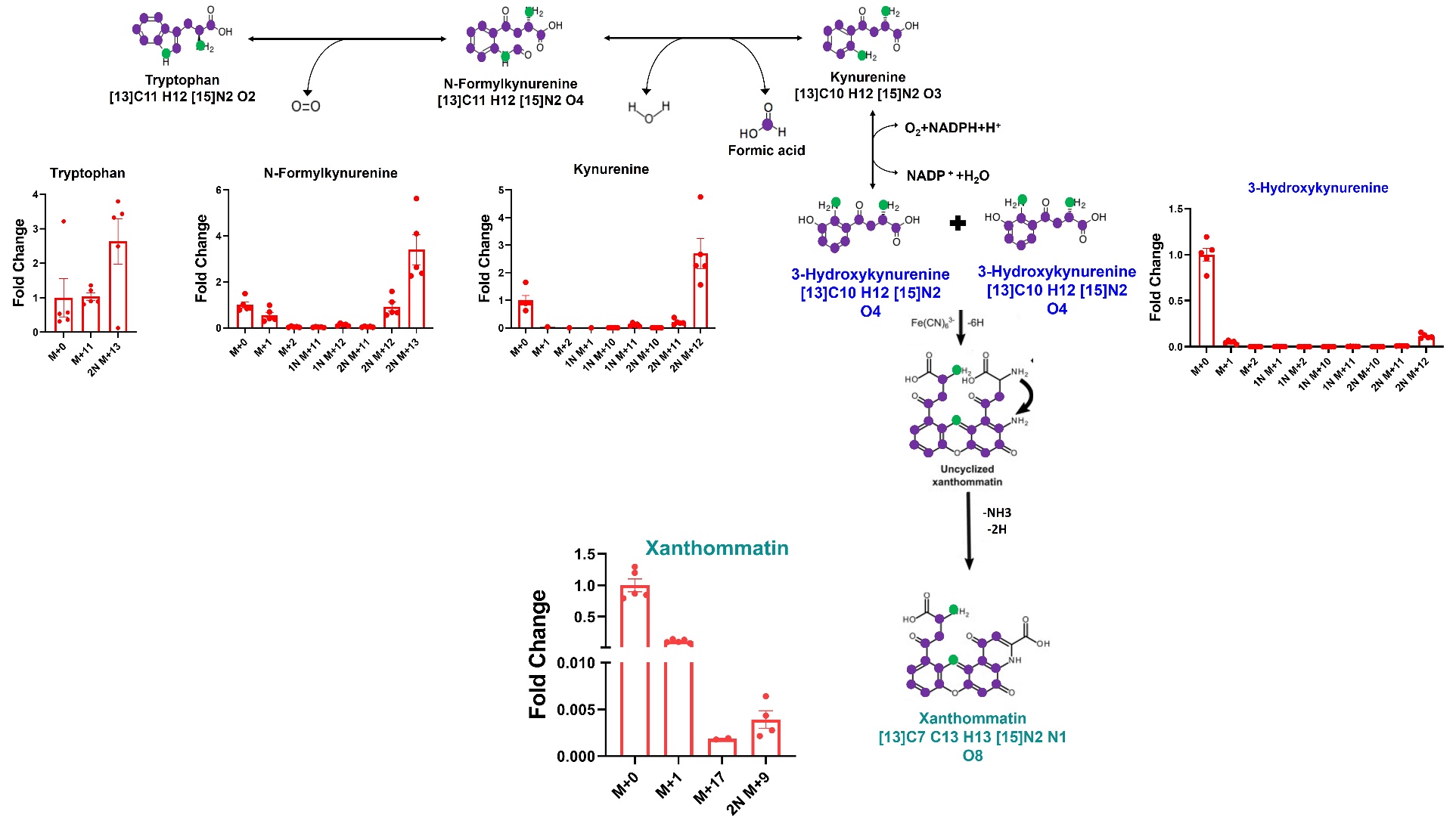
**

**Supplemental Figure 7:** Pathway possibilities for generation of xanthommatin from labeled tryptophan: Fold change for all detected isotopologues of each metabolite within the tryptophan-kynurenine-NAD^+^ pathway with respect to their m+0 isotopologue and the corresponding pathway illustration is shown. Purple dots represent labeled ^13^C and green dots represent labeled ^15^N. Data are shown as mean fold change ± SEM.

**
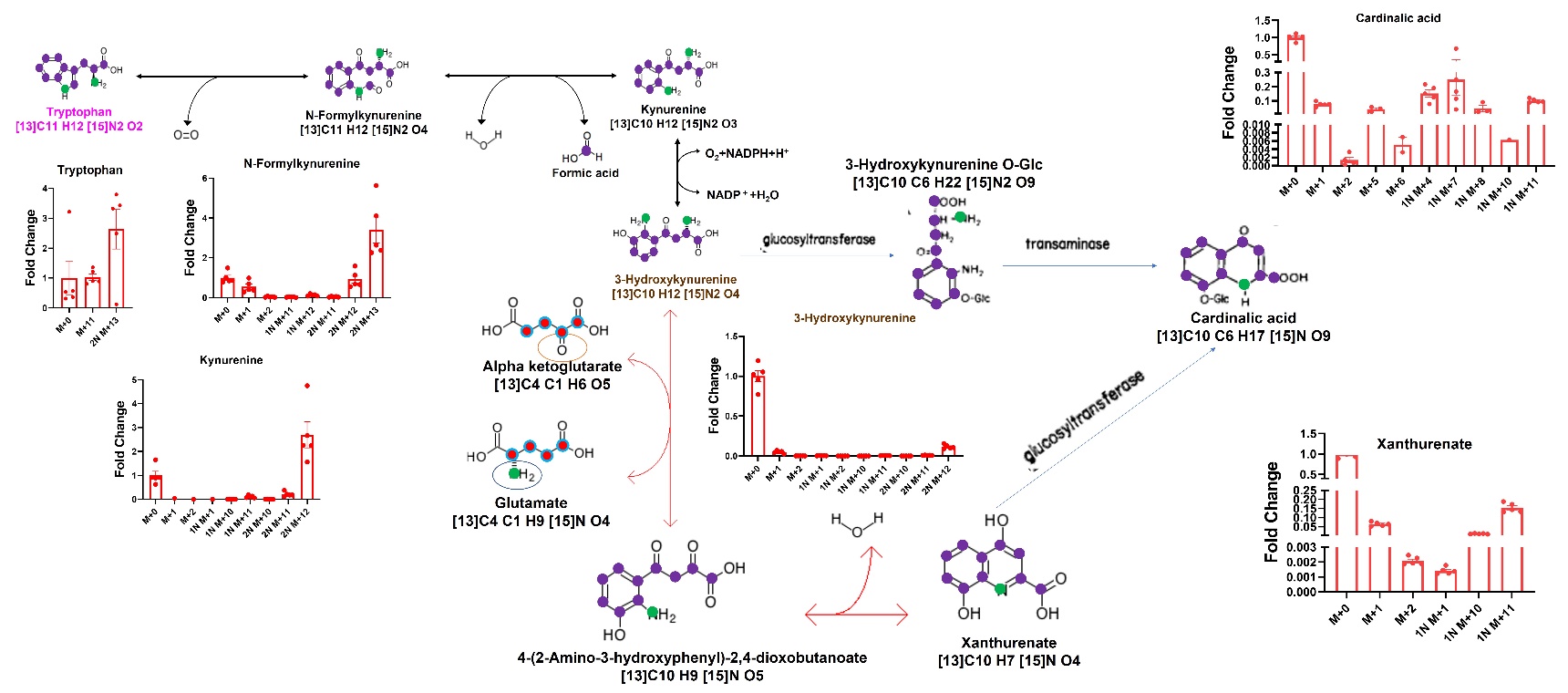
**

**Supplemental Figure 8: Pathway possibilities for generation of xanthurenate and cardinalic acid from labeled Tryptophan.** Fold change for all detected isotopologues of each metabolite within the tryptophan-kynurenine-NAD^+^ pathway with respect to their m+0 isotopologue and the corresponding pathway illustration is shown. Purple dots represent labeled ^13^C and green dots represent labeled ^15^N. Data are shown as mean fold change ± SEM.

**Supplemental figure 9.** Gene expression of KP enzymes in *Drosophila* which did not significantly change in response to one month of treatment with either 3-HK, α-MT, NAM, or α-MT+NAM. Data were analyzed using 1-way ANOVA with Tukey’s multiple comparisons test.

**Supplemental figure 10.**  Expression of longevity-associated genes in *Drosophila* which did not significantly change in response to one month of treatment with either 3-HK, α-MT, NAM, or α-MT+NAM. Data were analyzed using 1-way ANOVA with Tukey’s multiple comparisons test.

**Supplemental Methods: Details on SYBR Green Primers for Tryptophan Degradation Pathway Analysis and TaqMan Probes for Longevity Gene Detection:**

In this supplemental section, we provide a list of the primers we used in our experiments: the SYBR Green Primers for the analysis of the Tryptophan Degradation Pathway, and the TaqMan Probes for the detection of genes associated with longevity. The tables below list the sequences for the SYBR Green Primers (including product size and melting temperature), and the Assay ID for each of the TaqMan Probes. These resources were meticulously selected and utilized to ensure precise and reliable quantification of our target genetic material. We believe that these details will be of great value to researchers aiming to design similar studies in the future."

**SYBR Green Primers used for Tryptophan Pathway**

| **Name** | **Sequence (5' to 3')** | **Tm** |
| --- | --- | --- |
| *vermilion* (v) - F | GACGCTGGAGATTGTCAAGC | 61.1 |
| *vermilion* (v) - R | GAAGTCCATGAAGTCAAGCGG | 61 |
| *Tryptophan hydroxylase* - F | AGCATGACATAAACCTGGTGC | 60.6 |
| *Tryptophan hydroxylase* - R | GGCCTCCACGAAAAACTCATAAC | 61.7 |
| *Formamidase* - F | CTAGAGCACTTTGTTCGGGTG | 60.6 |
| *Formamidase* - R | GACGACCTTCTCCGTAGCG | 61.8 |
| Strain C1 nonfunctional mutant *cinnabar* (CN) - F | GAATCGTTAGCCAGGAGGTAAAC | 60.7 |
| Strain C1 nonfunctional mutant *cinnabar* (CN) - R | CGGTACTCGTACAAATCCACG | 60.5 |
| *Kynurenine aminotransferase* - F | CCTGGTCAATGCGCTATCCAA | 62.5 |
| *Kynurenine aminotransferase* - R | CCTGAGGTAATCAGTATGTCGC | 60 |
| *cardinal* - F | CAGGTCTCTCTGAAAATCCATCG | 60.4 |
| *cardinal* - R | CATGAACTGACCAAAAACGGC | 60.3 |
| *Phenoxazinone synthetase* - F | CAGGTCTCTCTGAAAATCCATCG | 60.4 |
| *Phenoxazinone synthetase* - R | CATGAACTGACCAAAAACGGC | 60.3 |

Primers for *rp49* expression were:

Forward, GTGAAGAAGCGCACCAAGCAC

Reverse, ACGCACTCTGTTGTCGATACCC

**TaqMan Probes for longevity**

| **Gene symbol** | **Gene aliases** | **Species** | **Assay ID** |
| --- | --- | --- | --- |
| *Foxo* | *forkhead box*, *sub-group O* | *Drosophila melanogaster* | Dm02140207_g1 |
| *Sirt2* | *Sirtuin 2* | *Drosophila melanogaster* | Dm02142601_g1 |
| *Indy* | *I'm not dead yet* | *Drosophila melanogaster* | Dm01793736_g1 |
| *Naam* | *Nicotinamide amidase* | *Drosophila melanogaster* | Dm02149964_m1 |
| *chico* | *CG5686-PA* | *Drosophila melanogaster* | Dm01803990_g1 |
| *sug* | *sugarbabe* | *Drosophila melanogaster* | Dm01823416_g1 |
| *HDAC1* | *Histone deacetylase 1* | *Drosophila melanogaster* | Dm01839959_g1 |
| *Mt2* | *Methyltransferase 2* | *Drosophila melanogaster* | Dm02370171_s1 |
| *inaE* | *inactivation no afterpotential E* | *Drosophila melanogaster* | Dm01847451_g1 |
| *Sirt1* | *Sirtuin 1* | *Drosophila melanogaster* | Dm01844783_g1 |
| *gig* | *gigas* | *Drosophila melanogaster* | Dm01823188_m1 |
| *RP49* | *RpL32* | *Drosophila melanogaster* | Dm02151827_g1 |

**References**:

1. Cervenka I, Agudelo LZ, Ruas JL. Kynurenines: Tryptophan's metabolites in exercise, inflammation, and mental health. *Science*. 2017;357(6349):10.1126/science.aaf9794.

2. Sang JH, King RC. Nutritional requirements of axenically cultured drosophila melanogaster adults. *J Exp Biol*. 1961;38(4):793-809.

3. Croset V, Schleyer M, Arguello JR, Gerber B, Benton R. A molecular and neuronal basis for amino acid sensing in the drosophila larva. *Scientific Reports*. 2016;6(1):34871. doi: 10.1038/srep34871.

4. Bender DA. Biochemistry of tryptophan in health and disease. *Mol Aspects Med*. 1983;6(2):101-197.

5. Liu L, Su X, Quinn WJ,3rd, et al. Quantitative analysis of NAD synthesis-breakdown fluxes. *Cell Metab*. 2018;27(5):1067-1080.e5.

6. Kanai M, Funakoshi H, Takahashi H, et al. Tryptophan 2,3-dioxygenase is a key modulator of physiological neurogenesis and anxiety-related behavior in mice. *Mol Brain*. 2009;2:8-8.

7. Canto C, Menzies KJ, Auwerx J. NAD(+) metabolism and the control of energy homeostasis: A balancing act between mitochondria and the nucleus. *Cell Metab*. 2015;22(1):31-53.

8. Fujigaki H, Saito K, Fujigaki S, et al. The signal transducer and activator of transcription 1alpha and interferon regulatory factor 1 are not essential for the induction of indoleamine 2,3-dioxygenase by lipopolysaccharide: Involvement of p38 mitogen-activated protein kinase and nuclear factor-kappaB pathways, and synergistic effect of several proinflammatory cytokines. *J Biochem*. 2006;139(4):655-662.

9. Chaves AC, Ceravolo IP, Gomes JA, Zani CL, Romanha AJ, Gazzinelli RT. IL-4 and IL-13 regulate the induction of indoleamine 2,3-dioxygenase activity and the control of toxoplasma gondii replication in human fibroblasts activated with IFN-gamma. *Eur J Immunol*. 2001;31(2):333-344.

10. Alberati-Giani D, Ricciardi-Castagnoli P, Kohler C, Cesura AM. Regulation of the kynurenine metabolic pathway by interferon-gamma in murine cloned macrophages and microglial cells. *J Neurochem*. 1996;66(3):996-1004.

11. Chiarugi A, Calvani M, Meli E, Traggiai E, Moroni F. Synthesis and release of neurotoxic kynurenine metabolites by human monocyte-derived macrophages. *J Neuroimmunol*. 2001;120(1-2):190-198.

12. Zunszain PA, Anacker C, Cattaneo A, et al. Interleukin-1beta: A new regulator of the kynurenine pathway affecting human hippocampal neurogenesis. *Neuropsychopharmacology*. 2012;37(4):939-949.

13. Matsuoka K, Kato K, Takao T, et al. Concentrations of various tryptophan metabolites are higher in patients with diabetes mellitus than in healthy aged male adults. *Diabetol Int*. 2016;8(1):69-75.

14. Schwarcz R, Bruno JP, Muchowski PJ, Wu HQ. Kynurenines in the mammalian brain: When physiology meets pathology. *Nat Rev Neurosci*. 2012;13(7):465-477.

15. Campesan S, Green EW, Breda C, et al. The kynurenine pathway modulates neurodegeneration in a drosophila model of huntington's disease. *Curr Biol*. 2011;21(11):961-966. doi: 10.1016/j.cub.2011.04.028 [doi].

16. Westbrook R, Chung T, Lovett J, et al. Kynurenines link chronic inflammation to functional decline and physical frailty. *JCI Insight*. 2020;5(16):e136091. doi: 10.1172/jci.insight.136091.

17. Wolfensberger M, Amsler U, Cuenod M, Foster AC, Whetsell WO,Jr, Schwarcz R. Identification of quinolinic acid in rat and human brain tissue. *Neurosci Lett*. 1983;41(3):247-252.

18. Pearson SJ, Reynolds GP. Determination of 3-hydroxykynurenine in human brain and plasma by high-performance liquid chromatography with electrochemical detection. increased concentrations in hepatic encephalopathy. *J Chromatogr*. 1991;565(1-2):436-440.

19. Parrott J,M., Redus L, O’Connor J,C. Kynurenine metabolic balance is disrupted in the hippocampus following peripheral lipopolysaccharide challenge. .

20. Ma S, Yim SH, Lee SG, et al. Organization of the mammalian metabolome according to organ function, lineage specialization, and longevity. *Cell Metab*. 2015;22(2):332-343.

21. Ooka H, Segall PE, Timiras PS. Histology and survival in age-delayed low-tryptophan-fed rats. *Mech Ageing Dev*. 1988;43(1):79-98.

22. Segall PE, Timiras PS. Patho-physiologic findings after chronic tryptophan deficiency in rats: A model for delayed growth and aging. *Mech Ageing Dev*. 1976;5(2):109-124.

23. De Marte ML, Enesco HE. Influence of low tryptophan diet on survival and organ growth in mice. *Mech Ageing Dev*. 1986;36(2):161-171.

24. van der Goot, A. T., Zhu W, Vazquez-Manrique RP, et al. Delaying aging and the aging-associated decline in protein homeostasis by inhibition of tryptophan degradation. *Proc Natl Acad Sci U S A*. 2012;109(37):14912-14917.

25. Oxenkrug GF, Navrotskaya V, Voroboyva L, Summergrad P. Extension of life span of drosophila melanogaster by the inhibitors of tryptophan-kynurenine metabolism. *Fly (Austin)*. 2011;5(4):307-309.

26. Oxenkrug GF. The extended life span of drosophila melanogaster eye-color (white and vermilion) mutants with impaired formation of kynurenine. *J Neural Transm (Vienna)*. 2010;117(1):23-26.

27. Balan V, Miller GS, Kaplun L, et al. Life span extension and neuronal cell protection by drosophila nicotinamidase. *J Biol Chem*. 2008;283(41):27810-27819. doi: S0021-9258(20)57709-0 [pii].

28. Fang EF, Hou Y, Lautrup S, et al. NAD(+) augmentation restores mitophagy and limits accelerated aging in werner syndrome. *Nat Commun*. 2019;10(1):5284-8. doi: 10.1038/s41467-019-13172-8.

29. Mouchiroud L, Houtkooper RH, Moullan N, et al. The NAD(+)/sirtuin pathway modulates longevity through activation of mitochondrial UPR and FOXO signaling. *Cell*. 2013;154(2):430-441. doi: S0092-8674(13)00755-1 [pii].

30. Mitchell SJ, Bernier M, Aon MA, et al. Nicotinamide improves aspects of healthspan, but not lifespan, in mice. *Cell Metab*. 2018;27(3):667-676.e4.

31. Ja WW, Carvalho GB, Mak EM, et al. Prandiology of drosophila and the CAFE assay. *Proc Natl Acad Sci U S A*. 2007;104(20):8253-8256. doi: 0702726104 [pii].

32. Mishra-Gorur K, Çağlayan AO, Schaffer AE, et al. Mutations in KATNB1 cause complex cerebral malformations by disrupting asymmetrically dividing neural progenitors. *Neuron*. 2014;84(6):1226-1239. doi: S0896-6273(14)01095-2 [pii].

33. Ramírez-Ortega D, Ramiro-Salazar A, González-Esquivel D, Ríos C, Pineda B, Pérez de la Cruz, Verónica. 3-hydroxykynurenine and 3-hydroxyanthranilic acid enhance the toxicity induced by copper in rat astrocyte culture. .

34. Okuda S, Nishiyama N, Saito H, Katsuki H. 3-hydroxykynurenine, an endogenous oxidative stress generator, causes neuronal cell death with apoptotic features and region selectivity. *J Neurochem*. 1998;70(1):299-307.

35. Presterud R, Deng WH, Wennerström AB, et al. Long-term nicotinamide riboside use improves coordination and eye movements in ataxia telangiectasia. *Mov Disord*. 2023. doi: 10.1002/mds.29645.

36. Brakedal B, Dölle C, Riemer F, et al. The NADPARK study: A randomized phase I trial of nicotinamide riboside supplementation in parkinson's disease. *Cell Metab*. 2022;34(3):396-407.e6. doi: 10.1016/j.cmet.2022.02.001.

37. Fang EF, Hou Y, Palikaras K, et al. Mitophagy inhibits amyloid-β and tau pathology and reverses cognitive deficits in models of alzheimer's disease. *Nat Neurosci*. 2019;22(3):401-412. doi: 10.1038/s41593-018-0332-9.

38. Savvateeva E, Popov A, Kamyshev N, et al. Age-dependent memory loss, synaptic pathology and altered brain plasticity in the drosophila mutant cardinal accumulating 3-hydroxykynurenine. *J Neural Transm (Vienna)*. 2000;107(5):581-601. doi: 10.1007/s007020070080.

39. Goldstein LE, Leopold MC, Huang X, et al. 3-hydroxykynurenine and 3-hydroxyanthranilic acid generate hydrogen peroxide and promote alpha-crystallin cross-linking by metal ion reduction. *Biochemistry*. 2000;39(24):7266-7275.

40. Eastman CL, Guilarte TR. Cytotoxicity of 3-hydroxykynurenine in a neuronal hybrid cell line. *Brain Res*. 1989;495(2):225-231.

41. Leipnitz G, Schumacher C, Dalcin KB, et al. In vitro evidence for an antioxidant role of 3-hydroxykynurenine and 3-hydroxyanthranilic acid in the brain. *Neurochem Int*. 2007;50(1):83-94. doi: 10.1016/j.neuint.2006.04.017.

42. Krause D, Suh H, Tarassishin L, et al. The tryptophan metabolite 3-hydroxyanthranilic acid plays anti-inflammatory and neuroprotective roles during inflammation: Role of hemeoxygenase-1. *The American Journal of Pathology*. 2011;179(3):1360-1372. doi: 10.1016/j.ajpath.2011.05.048.

43. Zhang L, Ovchinnikova O, Jönsson A, et al. The tryptophan metabolite 3-hydroxyanthranilic acid lowers plasma lipids and decreases atherosclerosis in hypercholesterolaemic mice. *Eur Heart J*. 2012;33(16):2025-2034. doi: 10.1093/eurheartj/ehs175.

44. Wang Q, Ding Y, Song P, et al. Tryptophan-derived 3-hydroxyanthranilic acid contributes to angiotensin II-induced abdominal aortic aneurysm formation in mice in vivo. *Circulation*. 2017;136(23):2271-2283. doi: 10.1161/CIRCULATIONAHA.117.030972.

45. Nainu F, Salim E, Asri RM, Hori A, Kuraishi T. Neurodegenerative disorders and sterile inflammation: Lessons from a drosophila model. *J Biochem*. 2019;166(3):213-221. doi: 10.1093/jb/mvz053.

46. Kounatidis I, Chtarbanova S, Cao Y, et al. NF-κB immunity in the brain determines fly lifespan in healthy aging and age-related neurodegeneration. *Cell Rep*. 2017;19(4):836-848. doi: S2211-1247(17)30482-5 [pii].

47. Jo J, Im SH, Babcock DT, et al. Drosophila caspase activity is required independently of apoptosis to produce active TNF/eiger during nociceptive sensitization. *Cell Death & Disease*. 2017;8(5):e2786. doi: 10.1038/cddis.2016.474.

48. Landis G, Shen J, Tower J. Gene expression changes in response to aging compared to heat stress, oxidative stress and ionizing radiation in drosophila melanogaster. *Aging (Albany NY)*. 2012;4(11):768-789. doi: 100499 [pii].

49. Lancaster GA, Sourkes TL. Effect of alpha-methyl-DL-tryptophan on tryptophan metabolism of musca domestica L. *Comp Biochem Physiol*. 1969;28(3):1435-1441. doi: 10.1016/0010-406x(69)90581-7 [doi].

50. Marygold SJ, Leevers SJ. Growth signaling: TSC takes its place. *Curr Biol*. 2002;12(22):785. doi: 10.1016/s0960-9822(02)01294-0.

51. Rogina B, Helfand SL. Sir2 mediates longevity in the fly through a pathway related to calorie restriction. *Proc Natl Acad Sci U S A*. 2004;101(45):15998-16003. doi: 10.1073/pnas.0404184101.

52. Kapahi P, Zid BM, Harper T, Koslover D, Sapin V, Benzer S. Regulation of lifespan in drosophila by modulation of genes in the TOR signaling pathway. *Curr Biol*. 2004;14(10):885-890. doi: 10.1016/j.cub.2004.03.059.

53. Whitaker R, Faulkner S, Miyokawa R, et al. Increased expression of drosophila Sir2 extends life span in a dose-dependent manner. *Aging (Albany NY)*. 2013;5(9):682-691. doi: 10.18632/aging.100599.

54. Kurnasov O, Goral V, Colabroy K, et al. NAD biosynthesis: Identification of the tryptophan to quinolinate pathway in bacteria. *Chem Biol*. 2003;10(12):1195-1204. doi: 10.1016/j.chembiol.2003.11.011.

55. MORAN JF, SOURKES TL. Induction of tryptophan pyrrolase by alpha-methyltryptophan and its metabolic significance in vivo. *J Biol Chem*. 1963;238:3006-3008.

56. Searles LL, Ruth RS, Pret AM, Fridell RA, Ali AJ. Structure and transcription of the drosophila melanogaster vermilion gene and several mutant alleles. *Mol Cell Biol*. 1990;10(4):1423-1431. doi: 10.1128/mcb.10.4.1423-1431.1990.

57. Huang W, Gong Z, Li J, Ding J. Crystal structure of drosophila melanogaster tryptophan 2,3-dioxygenase reveals insights into substrate recognition and catalytic mechanism. *J Struct Biol*. 2013;181(3):291-299. doi: 10.1016/j.jsb.2013.01.002.

58. Breda C, Sathyasaikumar KV, Sograte Idrissi S, et al. Tryptophan-2,3-dioxygenase (TDO) inhibition ameliorates neurodegeneration by modulation of kynurenine pathway metabolites. *Proc Natl Acad Sci USA*. 2016;113(19):5435. doi: 10.1073/pnas.1604453113.

59. Peng X, Zhao Z, Liu L, et al. Targeting indoleamine dioxygenase and tryptophan dioxygenase in cancer immunotherapy: Clinical progress and challenges. *Drug Des Devel Ther*. 2022;16:2639-2657. doi: 10.2147/DDDT.S373780.

60. Mackay TF, Richards S, Stone EA, et al. The drosophila melanogaster genetic reference panel. *Nature*. 2012;482(7384):173-178. doi: 10.1038/nature10811 [doi].

61. Jia H, Li X, Gao H, et al. High doses of nicotinamide prevent oxidative mitochondrial dysfunction in a cellular model and improve motor deficit in a drosophila model of parkinson's disease. *J Neurosci Res*. 2008;86(9):2083-2090. doi: 10.1002/jnr.21650.

62. Gabrawy MM, Campbell S, Carbone MA, et al. Lisinopril preserves physical resilience and extends life span in a genotype-specific manner in drosophila melanogaster. *J Gerontol A Biol Sci Med Sci*. 2019;74(12):1844-1852. doi: 10.1093/gerona/glz152 [doi].

63. Komatsu T, Park S, Hayashi H, Mori R, Yamaza H, Shimokawa I. Mechanisms of calorie restriction: A review of genes required for the life-extending and tumor-inhibiting effects of calorie restriction. *Nutrients*. 2019;11(12):3068. doi: 10.3390/nu11123068. doi: 10.3390/nu11123068.

64. Piper MDW, Blanc E, Leitão-Gonçalves R, et al. A holidic medium for drosophila melanogaster. *Nat Methods*. 2014;11(1):100-105. doi: 10.1038/nmeth.2731.

65. Consoulas C, Restifo LL, Levine RB. Dendritic remodeling and growth of motoneurons during metamorphosis of drosophila melanogaster. *J Neurosci*. 2002;22(12):4906-4917. doi: 22/12/4906 [pii].

66. Ikeda K, Koenig JH. Morphological identification of the motor neurons innervating the dorsal longitudinal flight muscle of drosophila melanogaster. *J Comp Neurol*. 1988;273(3):436-444. doi: 10.1002/cne.902730312 [doi].

67. Ryglewski S, Vonhoff F, Scheckel K, Duch C. Intra-neuronal competition for synaptic partners conserves the amount of dendritic building material. *Neuron*. 2017;93(3):632-645.e6. doi: S0896-6273(16)31042-X [pii].

68. Vonhoff F, Kuehn C, Blumenstock S, Sanyal S, Duch C. Temporal coherency between receptor expression, neural activity and AP-1-dependent transcription regulates drosophila motoneuron dendrite development. *Development*. 2013;140(3):606-616. doi: 10.1242/dev.089235 [doi].

69. Vonhoff F, Duch C. Tiling among stereotyped dendritic branches in an identified drosophila motoneuron. *J Comp Neurol*. 2010;518(12):2169-2185. doi: 10.1002/cne.22380 [doi].

70. Heinrich L, Ryglewski S. Different functions of two putative drosophila α(2)δ subunits in the same identified motoneurons. *Sci Rep*. 2020;10(1):13670-8. doi: 10.1038/s41598-020-69748-8 [doi].

71. Vonhoff F, Williams A, Ryglewski S, Duch C. Drosophila as a model for MECP2 gain of function in neurons. *PLoS One*. 2012;7(2):e31835. doi: 10.1371/journal.pone.0031835 [doi].

72. Hutchinson KM, Vonhoff F, Duch C. Dscam1 is required for normal dendrite growth and branching but not for dendritic spacing in drosophila motoneurons. *J Neurosci*. 2014;34(5):1924-1931. doi: 10.1523/JNEUROSCI.3448-13.2014 [doi].

73. Sanyal S. Genomic mapping and expression patterns of C380, OK6 and D42 enhancer trap lines in the larval nervous system of drosophila. *Gene Expr Patterns*. 2009;9(5):371-380. doi: 10.1016/j.gep.2009.01.002 [doi].

74. Boerner J, Godenschwege TA. Whole mount preparation of the adult drosophila ventral nerve cord for giant fiber dye injection. *J Vis Exp*. 2011;(52):3080. doi(52):10.3791/3080. doi: 3080 [pii].
